# Apicidin F: Characterization and Genetic Manipulation of a New Secondary Metabolite Gene Cluster in the Rice Pathogen *Fusarium fujikuroi*

**DOI:** 10.1371/journal.pone.0103336

**Published:** 2014-07-24

**Authors:** Eva-Maria Niehaus, Slavica Janevska, Katharina W. von Bargen, Christian M. K. Sieber, Henning Harrer, Hans-Ulrich Humpf, Bettina Tudzynski

**Affiliations:** 1 Westfälische Wilhelms-Universität Münster, Institut für Biologie und Biotechnologie der Pflanzen, Münster, Germany; 2 Westfälische Wilhelms-Universität Münster, Institut für Lebensmittelchemie, Münster, Germany; 3 Helmholtz Zentrum München (GmbH), Institut für Bioinformatik und Systembiologie, Neuherberg, Germany; Seoul National University, Republic Of Korea

## Abstract

The fungus *F. fujikuroi* is well known for its production of gibberellins causing the ‘bakanae’ disease of rice. Besides these plant hormones, it is able to produce other secondary metabolites (SMs), such as pigments and mycotoxins. Genome sequencing revealed altogether 45 potential SM gene clusters, most of which are cryptic and silent. In this study we characterize a new non-ribosomal peptide synthetase (NRPS) gene cluster that is responsible for the production of the cyclic tetrapeptide apicidin F (APF). This new SM has structural similarities to the known histone deacetylase inhibitor apicidin. To gain insight into the biosynthetic pathway, most of the 11 cluster genes were deleted, and the mutants were analyzed by HPLC-DAD and HPLC-HRMS for their ability to produce APF or new derivatives. Structure elucidation was carried out be HPLC-HRMS and NMR analysis. We identified two new derivatives of APF named apicidin J and K. Furthermore, we studied the regulation of APF biosynthesis and showed that the cluster genes are expressed under conditions of high nitrogen and acidic pH in a manner dependent on the nitrogen regulator AreB, and the pH regulator PacC. In addition, over-expression of the atypical pathway-specific transcription factor (TF)-encoding gene *APF2* led to elevated expression of the cluster genes under inducing and even repressing conditions and to significantly increased product yields. Bioinformatic analyses allowed the identification of a putative Apf2 DNA-binding (“Api-box”) motif in the promoters of the *APF* genes. Point mutations in this sequence motif caused a drastic decrease of APF production indicating that this motif is essential for activating the cluster genes. Finally, we provide a model of the APF biosynthetic pathway based on chemical identification of derivatives in the cultures of deletion mutants.

## Introduction

Fungi are well known for their ability to produce a great range of natural products, the so called secondary metabolites (SMs). These compounds are structurally diverse, low molecular mass molecules that are not essential for the growth and survival of the producing organism, but instead are thought to increase its fitness or to decrease the fitness of surrounding organisms [1]. The increasing number of sequenced fungal genomes has revealed an enormous potential of fungi to produce many more SMs than originally expected. Most of these new SMs are only predicted by bioinformatics analysis of putative SM gene clusters in sequenced genomes. Unfortunately, many of these potential SM gene clusters show little or no expression under typical laboratory conditions, and therefore the potential new SMs are either not produced, or are present at levels that are too low to be detected by standard methods [2]. In some cases, the production of such cryptic or silent SM-derived gene clusters has been successfully induced by genetic manipulation [1].

**Figure 1:**
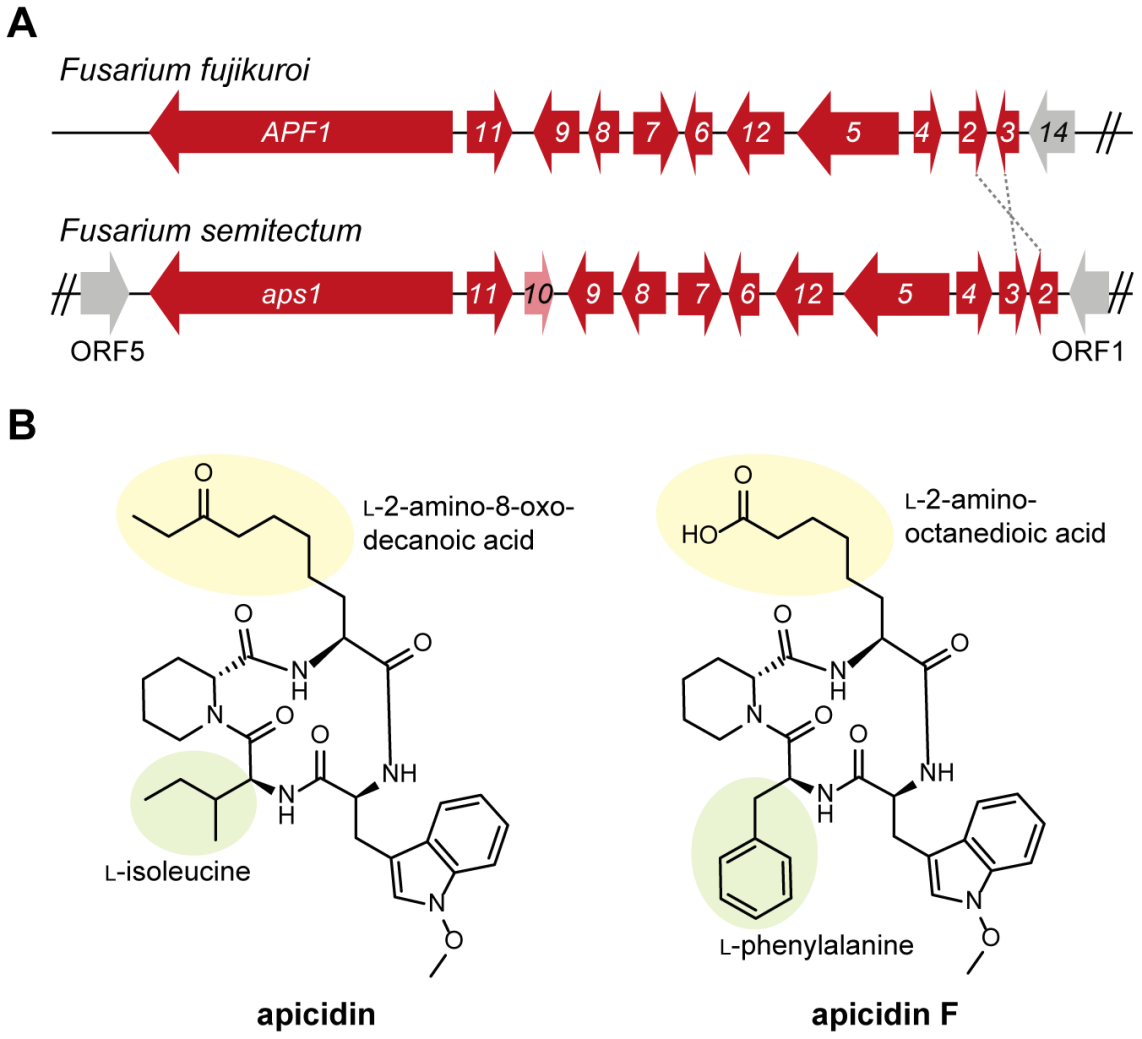
Cluster of apicidin F in *Fusarium fujikuroi* and apicidin in *F. semitectum* and their corresponding structures. (A) The apicidin F gene cluster (*APF1*-*APF12*) is located at the terminal part of chromosome I, thus it has only one border gene (*FFUJ_00014*). The arrows indicate the direction of transcription. In comparison to *F. semitectum*, *F. fujikuroi* is missing *aps10* and *aps2*/*APF2* & *aps3*/*APF3* are orientated in an opposite manner. (B) Apicidin is produced by *F. semitectum* [14]. Apicidin F is produced by *F. fujikuroi* [10].

The genus *Fusarium* comprises broadly distributed, plant-pathogenic species that are able to infect economically important crops leading to huge losses. The rice pathogen *Fusarium fujikuroi* is one of the first described plant-pathogenic *Fusarium* species and is widespread in all rice-growing countries of the world. It causes the ‘bakanae’ (foolish seedling) disease due to its ability to produce and secrete gibberellins (GAs), a family of plant hormones [3], [4]. In addition, *F. fujikuroi* produces some other well-known SMs, particularly pigments like bikaverin and fusarubins, and mycotoxins, such as fusarins and fusaric acid [5]–[8]. However, the recently sequenced genome of *Fusarium fujikuroi* revealed altogether 45 gene clusters, mostly with unknown function (cryptic), indicating an enormous genetic potential to produce new SMs. One of them is a novel non-ribosomal peptide synthetase (NRPS) gene cluster with NRPS31 as key enzyme [9]. The product of this cluster has been recently elucidated in *F. fujikuroi* by NMR and MS analyses and was designated apicidin F (APF) [10]. APF belongs to the group of cyclic tetrapeptides, the apicidins, which are built up of four amino acids. The founding member of the group, apicidin (APS), contains the amino acids *N*-methoxy-l-tryptophan, l-isoleucine, d-pipecolic acid and l-2-amino-8-oxodecanoic acid (Aoda) and was identified in *Fusarium pallidoroseum*, renamed *F. semitectum* and in *Fusarium sambucinum* (KCTC 16677) [11]–[13]. Meanwhile, seven additional APS derivatives have been identified either as synthetic analogs or naturally occurring compounds [14]–[16]. In contrast to APS, APF contains l-phenylalanine instead of isoleucine and l-2-aminooctanedioic acid instead of Aoda, while the other two amino acids, *N*-methoxy-l-tryptophan and d-pipecolic acid, are common in APS and APF (Fig. 1A) [10]. APS is known to be a histone deacetylase (HDAC) inhibitor and a potential anticancer agent [17]. Due to its HDAC-inhibiting activities, APS has antiprotozoal effects against the murine malaria species *Plasmodium berghei* and *P. falciparum* [12]. Several structure-activity relationship studies revealed a large impact of the amino acid composition on the HDAC inhibitory activity [15], [16], [18]–[20]. Because of its variation in the Aoda side chain and an additional amino acid substitution (l-phenylalanine instead of isoleucine), the new APS derivative APF was tested for antimalarial activity and showed *in vitro* activity against *P. falciparum* comparable to APS [10].

Although the production of APS by *F. semitectum* was well known for more than ten years, the corresponding APS biosynthesis gene cluster has been identified only recently [14]. In the genome sequence of *F. fujikuroi*, we detected a similar gene cluster at the far end of chromosome I which is surprisingly absent from any other genome of the highly related *Fusarium* species of the *Gibberella fujikuroi* species complex, such as *F. verticillioides*, *F. mangiferae* and *F. circinatum* [9]. For better differentiation between the gene clusters in *F. semitectum* and *F. fujikuroi*, we renamed the *F. fujikuroi* genes *APF* for **ap**icidin **F** biosynthetic genes.

In this work, we studied the function of most of the APF cluster genes by gene replacement and subsequent high-performance liquid chromatography (HPLC) coupled to high resolution mass spectrometry (HRMS)-based identification of products accumulated in the cultures of single deletion mutants. We were able to elucidate the structures of two new derivatives, apicidin J and apicidin K. Gene expression studies and product measurements revealed that APF production in *F. fujikuroi* is induced under nitrogen-sufficient and acidic pH conditions by the global regulators AreB and PacC, respectively. In addition, the presence of the pathway-specific TF Apf2 is essential for *APF* gene expression. Over-expression of the Apf2-encoding gene resulted in elevated cluster gene expression and significantly increased product yields even under repressing nitrogen-limiting conditions. The binding motif for Apf2 that was indicated by bioinformatics has now been proved experimentally by promoter mutagenesis.

## Material and Methods

### Strains and media

For generation of over-expression and deletion mutants the wild type (WT) strain *Fusarium fujikuroi* IMI58289 (Commonwealth Mycological Institute, Kew, UK) was applied. For expression studies the following mutants were used: Δ*AREA* [21], Δ*AREB* [22], Δ*GLN1* [23] Δ*VEL1*, Δ*VEL2*, Δ*LAE1* [24], Δ*PACC* [25], and Δ*CPR* deletion mutants [26].

For cultivation of the strains, a 300-mL Erlenmeyer flask with 100 mL Darken medium (DVK) [27] was inoculated with the respective strain and then shaken at 180 rpm at 28°C for 72 h in the dark. The main culture was also grown in a 300-mL flask and inoculated with 500 µL of the DVK. This culture consists of 100 mL synthetic medium (Imperial Chemical Industries, UK, ICI) [28] with different amounts of nitrogen: 6 or 120 mM NaNO_3_ or 6 or 60 mM glutamine, respectively. On a rotary shaking incubator, the culture was shaken for 2–6 days at 180 rpm in the dark. The culture broth was harvested and the mycelium was used for extraction of APF analogs and RNA isolation. Transformation of *F. fujikuroi* and DNA isolation was done as described [9]. The pH shift experiments with the WT and the Δ*PACC* mutant were performed as described [29].

### Chemicals

The solvents and reagents were purchased from Sigma-Aldrich (Deisenhofen, Germany), VWR (Darmstadt, Germany) in gradient or analytical grade. A Milli-Q Gradient A10 system (Millipore, Schwalbach, Germany) was used for the generation of water used during chromatography and extraction. APS for cell culture studies was purchased from Enzo lifesciences (Farmindale, New York). l- and dl-proline were from Sigma-Aldrich (Deisenhofen, Germany). The DMEM medium and fetal calf serum were purchased from Biochrom AG (Berlin, Germany).

### Standard molecular methods

Standard techniques were used for DNA and RNA analysis [30]. By grinding the isolated lyophilized mycelium into a powder, it can then be used for extraction of DNA [31]. For Southern blot analysis, digestion of this DNA was done with the respective restriction enzymes (Fermentas GmbH, St. Leon-Rot, Germany). These approaches were fractionated on a 1% (w/v) agarose gel and transferred by downward blotting onto a Nytran nylon transfer membrane (Whatman Inc., Sanford, ME, USA) [32]. In accordance with the protocol of Sambrook *et al*., the probes were labeled with ^32^P and then used for the random oligomer-primer method [30]. Southern blot analyses of Δ*APF1*, Δ*APF2*, Δ*APF3*, Δ*APF6*, Δ*APF9* and Δ*APF11* are depicted in Fig. S4–S9 in File S1. The RNAgents total RNA isolation kit (Promega GmbH, Mannheim, Germany) was used for the isolation of RNA based on the harvested fungal mycelium. Samples of 20 µg were used for the separation on a 1% (w/v) agarose gel containing 1% (v/v) formaldehyde [30]. The northern blot hybridizations were done according to Church and Gilbert [33].

All primers used in this study are listed in Table S1. Polymerase chain reactions (PCR) contained 5 pmol of each primer, 200 nM dNTPs, 25 ng DNA and 1 unit of BioThermDNA polymerase (GeneCraft GmbH, Lüdinghausen, Germany) and were started at 94°C for 4 min and continued for 36 cycles at 94°C for 1 min, 1 min at 56–60°C, 1–2 min at 70°C and the last step for 10 min at 70°C.

### Plasmid construction

For generating deletion mutants, the putative flanking regions of the desired gene were amplified using the primer pair “gene”-3R and “gene”-3F for the 3′ flank and “gene”-5R and “gene”-5F for the 5′ flank. The hygromycin (*HPH*) and nourseothricin (*NAT*) resistance cassettes were amplified using the hph-F and hph-R primers. Both *HPH* and *NAT* are under the control of the *TRPC* promoter from *A. nidulans* and were amplified based on the vectors pCSN44 [34] and pZPnat1 (GenBank Accession No. AY631958.1), respectively. The gene replacement vectors pΔ*APF1* (*HPH*), pΔ*APF2* (*HPH*), pΔ*APF3* (*HPH*), pΔ*APF6* (*HPH*), pΔ*APF9* (*HPH*) and pΔ*APF11* (*NAT*) were created using the yeast recombinational system [35]–[37].

For cloning the *GFP-APF2* fusion construct, *APF2* was amplified without stop codon using the primer pair GFP_APF2_F and GFP_APF2_R as well as proof-reading Phusion polymerase (Finnzymes, Thermo Fisher Scientific). The obtained fragment was transformed into *S. cerevisiae* together with the NcoI-restricted plasmid pNDN-OGG containing the nourseothricin resistance cassette driven by the *TRPC* promoter and the *GFP* gene. The *APF2*-*GFP* fusion was driven by the strong *A. nidulans OLIC* promoter [35]. The created vector was transformed into the deletion mutant of *APF2* (Δ*APF2*). For generating the over-expression mutant OE::*APF2*, the constructed vector *APF2*-*GFP* was transformed into the WT.

For creating the point mutation mutants (P-Mut1 and P-Mut2), the promoter region of *APF11*/*APF1* and the coding region of *APF1* were amplified using the primer pairs Prom_apf1_R/Papf1_mut1/2_F and Prom_apf1_F/Papf1_mut1/2_R (Table S1). Mutations were introduced into the putative DNA-binding motif (5′-TGACGAGA-3′): P_Mut1 5′-GGAAGAGA-3′ and P_Mut2 5′-TGACGCGA-3′. The obtained fragments and a SacII/SpeI-restricted pNDH-OGG vector (containing the *HPH* cassette driven by the *A. nidulans TRPC* promoter) were cloned into the yeast strain FY834 as described above. Afterwards, sequencing was done with the BigDye Terminator v3.1 Cycle Sequencing Kit and the ABI Prism 3730 Genetic Analyzer (Applied Biosystems, Foster City, CA, USA).

The yeast DNA was isolated by using the yeast plasmid isolation kit (SpeedPrep, DualsystemsBio Tech) and transformed into *E. coli* TOP 10 (Invitrogen). For extraction of the plasmid DNA from *E. coli*, the GeneJet Plasmid Miniprep Kit (Thermo Fisher Scientific, St. Leon-Rot, Germany) was used. The deletion fragments were amplified by PCR with 1 µL of the extracted yeast DNA as template and the primer pair “gene”-5F and “gene”-3R using the TaKaRa polymerase kit (TaKaRa Biotechnology (Dalian) Co., LTD, Japan).

### Fungal transformations

*F. fujikuroi* transformations were carried out as described [38]. About 10^7^ protoplasts were transformed with the PCR product for generating deletion mutants and with 10 µg of the vector for gaining over-expression, point mutated and *GFP* mutants, as described before. After regeneration for 4–5 days at 28°C in the dark on a complete regeneration agar (0.05% yeast extract and 0.7 M sucrose) containing either 100 µg/mL hygromycin B (Calbiochem, Darmstadt, Germany) or 100 µg/mL nourseothricin (Werner-Bioagents, Jena, Germany), the transformants were controlled by diagnostic PCR and Southern blot. To verify homologous integration of gene replacement fragments by diagnostic PCR, the following primer pair was used: “gene”-5F-diag/pCSN44-hph-trpC-T and “gene”-3R-diag/pCSN44-hph-P2. For checking the absence or the presence of the WT gene, the pair “gene”-R/“gene”-F was used. From a pool of about 20–30 transformants for each transformation experiment, the following transformants were found to have homologous integrations of the respective replacement cassette: for Δ*APF1* two mutants (T3, T4), for Δ*APF2* three mutants (T3, T5, T9), for Δ*APF3* three mutants (T1, T3, T4), for Δ*APF6* three mutants (T1, T2, T4), for Δ*APF9* three mutants (T2, T4, T8), and for Δ*APF11* two mutants (T4, T5).

### Microscopy of the GFP-TF Apf2

For fluorescence microscopy an AxioImager M1 (Carl Zeiss MicroImaging GmbH) was used. Brightfield images were made with differential interference contrast (DIC) microscopy. GFP fluorescence was analyzed with the filter set 38 (excitation BP 470/40, beam splitter FT 495, emission BP 525/50). The Zeiss AxioCam MRm camera was taken for images. The Axiovison Rel 4.8 software package was used for processing the data. 10 µL of a dilution (1∶1000) of the fluorescent dye Hoechst 33342 were used for staining the nuclei [39]. Examination of the stained nuclei was carried out with the filter set 49 DAPI shift free (excitation G 365, beam splitter FT 395, emission BP 445/50).

### Analysis of APF and its derivatives by HPLC-DAD and HPLC-HRMS analysis

APF was extracted from the harvested mycelium after incubation in ICI medium for 3–6 days. Therefore, 0.1 g of the lyophilized mycelium was extracted for 2 h with 1.5 mL ethylacetate/methanol (3∶2, v/v). 0.75 mL of the supernatant were evaporated and re-suspended in 1.5 mL 30% acetonitrile (ACN) (v/v). The amounts of APF were measured by high-performance liquid chromatography-diode array detector (HPLC-DAD) analysis at a wavelength of 280 nm. A Merck-Hitachi System (Merck KGaA, Darmstadt, Germany) was used with an autosampler (L-7200), a Diode Array Detector (L-245) and a gradient pump (L-7100). The samples were separated on a LiChrospher 100 RP-18 column (5 µm, 250 mm×4 mm, Merck KGaA). The following conditions were used: solvent A ACN with 1% formic acid (v/v), solvent B 1% formic acid (v/v). The gradient was from 30% A to 45% A in 10 min, then for 15 min up to 50% A and followed by column flushing for 5 min at 100% A. Equilibration at the starting condition of 30% A was carried out for 3 min. The injection volume was 80 µL and the flow rate 1 mL/min. EZChrom *Elite* Version 3.3.2 SP1 (Scientific Software, Inc.) was used for analyzing the data. The standard APF was used for compound identification. The concentration of the standard was 0.1 µg/µL dissolved in 30% ACN.

The HPLC-HRMS analysis on an LTQ Orbitrap XL-mass spectrometer uses a method previously described for the analysis of APF [9] with minor changes: capillary temperature 225°C, vaporizer temperature 275°C. Instead of the gradient described by Wiemann et al., 2013, the following shorter gradient was applied. Solvent A: methanol with 1% formic acid (v/v), solvent B: 1% formic acid (v/v). The gradient was from 10% A to 100% A in 20 min followed by column flushing at 100% A for 5 min and equilibration to the starting conditions for 5 min at 40°C. Additionally to the positive ionization mode used for HRMS analysis, the ion trap was used for detection of the deprotonated molecular ions in the negative ionization mode to determine the molecular mass of the biosynthetic analogs. Source voltage was −3.0 kV, capillary voltage −35 V and tube lens −110 V for the negative mode. 10 µL of the culture filtrate or the mycelium extract were injected. For semi-quantitative estimation of the produced amount of APF in the mutants P_Mut1 and P_Mut2, 10 µL of a 1 µg/mL APS stock solution was added as internal standard to 90 µL of the mycelium extract prepared for HPLC analysis. The peak area of APF was normalized to the internal standard. Therefore, the extracted ion chromatograms of [M+H]^+^ of APF (646.3235±0.0032) and APS (624.3756±0.0032) were used.

### Isolation of apicidin J and apicidin K

For the isolation of apicidin J and apicidin K, the Δ*APF3*/OE::*APF2* and the Δ*APF9*/OE::*APF2* mutants, respectively, were grown for three days in 60 mM glutamine. The culture filtrates were extracted on a Strata C18-E (55 µm, 70 Å) 10 g/60 mL SPE column (Phenomenex, Aschaffenburg, Germany) as previously described [9]. In detail, the column was pretreated with 50 mL methanol and 50 mL water. Approximately 1 L of the culture filtrate was loaded onto the column. After application of about 500 mL culture filtrate, the column was washed with 50 mL water to prevent plugging of the column with salts and sugars. When the column was loaded completely, it was washed with 100 mL water followed by washing with 20% and 40% methanol/water (v/v). Apicidin J was eluted with 80% methanol/water (v/v), Apicidin K was eluted with 80% methanol/water (v/v) after washing with 60% methanol/water (v/v). All steps were performed under vacuum. The fractions were evaporated to dryness on a rotary evaporator at 40°C. Further purification was performed on a preparative HPLC-UV. The first preparative HPLC-UV run was the same as for APF isolation [9] except for another HPLC system used. The preparative HPLC-UV was carried out on a Jasco system (Jasco PU-2087 pump coupled to Jasco UV-2075-detector, Jasco, Groβ-Umstadt, Germany).

The dried fractions of the SPE clean-up were dissolved in a preferably small volume of the starting conditions, and about 1.8 mL were injected per run. Apicidin J elutes at about 14 min and apicidin K at about 21 min. After the preparative HPLC, the apicidin J fraction was further purified in a second run using the same 250×10.0 mm Varian Microsorb 100–5 C_18_ column with a 10.0×10.0 mm Gemini C_6_-Phenyl-guard column. Solvent A was 5% THF in methanol (v/v), solvent B was water. The flow rate was 3.5 mL/min. The run was isocratic at 65% A. The UV-detector was set to 254 nm. The purity was determined with an Evaporative Light Scattering Detector (ELSD). The final yield of apicidin J was about 2 mg out of 1 L culture filtrate with a purity of ≥98% (HPLC-ELSD).

The apicidin K fraction was finally purified on a Shimadzu-DAD with DGU-20A_3_ – degasser, SIL-20AF – autosampler, 2 LC-10ATvp pumps, SPD- M20A – DAD-detector, CBM-20A –controle module, CTO-10ASvp – column oven. The used column was a 250×4.6 mm Gemini C_18_ 5 µm column with a 4.0×3.0 mm guard column of the same material. The flowrate was 1 mL/min and the wavelength was set to 254 nm. Injection volume was 50 µL. The sample was dissolved in a low amount of the starting conditions. The separation was isocratic at 70% A and 40°C. The purity was determined with an ELSD. The final yield of apicidin K was about 5.5 mg out of 1.5 L culture filtrate with a purity of ≥95% (HPLC-ELSD).

The system used for the determination of the purity was a Shimadzu-UV-ELSD (DGU-20A_3_ – degasser, SIL-20A – autosampler, 2 LC-20AT pumps, SPD-20UV – UV-detector, CBM-20A – control module, ELSD-LT, software LCsolution). The column used was a 250×4.6 mm Gemini C_18_ 5 µm column with a 4.0×3.0 mm guard column of the same material. The flowrate was 1 mL/min. The ELSD was set to 40°C and 250 kPa with compressed air. The UV detector was set to 275 nm. Solvent A was 1% formic acid in methanol (v/v), solvent B was 1% formic acid in water (v/v). The gradient was from 30% to 100% A in 30 min, 100% A for 5 min and equilibration to 30% A for 10 min. The purity of the isolated compounds was tested with an about 50 µg/mL solution in 10% methanol with an injection volume of 15 µL and compared to a 10% methanol blank solution.

### Structure elucidation of apicidin J and apicidin K

The structure of apicidin J could not be elucidated using nuclear magnetic resonance (NMR)-spectroscopy because the isolated quantity was insufficient for full NMR-data. Instead, a method was applied that combined hydrolysis, identification and derivatization of the amino acids, as well as a partial hydrolysis with a vaporizer temperature of 300°C and a capillary temperature of 250°C followed by sequencing of the resulting di- and tripeptides, as previously described for APF [10]. The hydrolysis of the cyclic tetrapeptides was carried out using 5% thioglycolic acid in 6 M hydrochloric acid (HCl) to prevent tryptophan degradation as previously described [10]. Amino acid analysis was performed as described in von Bargen et al., 2013 using proline, phenylalanine, tryptophan and 2-aminooctanedioic acid as reference compounds. Marfey's derivatization of the amino acids was described in von Bargen et al., 2013 with a modified gradient for better separation of d- and l-proline [10]. Solvent A was 1% formic acid in methanol (v/v), solvent B was 1% formic acid (v/v). The gradient was 20% A for 2 min, followed by a gradient up to 45% A in 15 min, after 1 min at 45% the column was equilibrated to starting conditions of 20% A for 10 min. Instead of pipecolic acid, d- and dl-proline were used (Fig. S11 in File S1). Partial hydrolysis and analysis of the di- and tripeptides was also adapted from APF with a slight modification in the MS-parameters [10]. Capillary temperature was 250°C and vaporizer temperature was 300°C (Fig. S12 in File S1).

The structure of apicidin K was elucidated using NMR-spectroscopy and hydrolysis followed by Marfey's derivatization (Fig. S13–S18 in File S1). For NMR, apicidin K was dissolved in C_5_D_5_N and ^1^H-, ^13^C-spectra as well as H, H-correlated spectroscopy (H, H-COSY), heteronuclear single quantum coherence (HSQC)- and heteronuclear multiple bond correlation (HMBC)-experiments were performed. The pulse programs were taken from the software library. The spectra were recorded on a 400 MHz Bruker DPX 400 NMR spectrometer (Bruker, Rheinstetten, Germany). MestReNova 7.1.1 (Mestrelab Research S.L., Santiago de Compostela, Spain) was used for data evaluation. Hydrolysis and Marfey's derivatization were performed as described above without modification of the gradient from [10]. Since 2-amino-8-hydroxyoctanoic acid is not commercially available, the stereochemistry of this amino acid could not be determined finally (Fig. S17; S18 in File S1). NMR-data for apicidin K are listed in Table S2 in File S1. The spectra are depicted in Fig. S13-16 in File S1.

### Cytotoxicity assays for APF

For cell culture studies, the human liver hepatocellular carcinoma cell line Hep G2 was used. About 1×10^4^ cells were seeded in 96-well plates in Dulbeccos Modified Eagle Medium supplemented with 10 mM 4-(2-hydroxyethyl)-1-piperazineethanesulfonic acid (HEPES), 100 IU/mL penicillin, 100 µg/mL streptomycin, 2 mM L-glutamine and 10% (v/v) fetal calf serum (FCS) and cultivated in 5% CO_2_ (v/v) at 37°C for 24 h. After starvation for 24 h in medium without serum, APS or APF were applied in concentrations from 1 ng/mL to 100 µg/mL. The substances were incubated for 48 h. The viability of the cells was determined using the Cell Counting Kit-8 (CCK-8)-assay which was performed as described previously [40].

### Statistical data evaluation

For statistical data evaluation, the software SigmaPlot 12 (Systat Software Inc., San Jose, California) was used to perform the analysis of variance (ANOVA) with the Tukey post-hoc test with p≤0.05. Additionally, SigmaPlot 12 was used to calculate the IC_50_-values.

### Determination of cluster specific cis-regulatory motifs

We applied Phylocon, an algorithm for finding conserved motifs in orthologs [41], and the de-novo methods Meme [42] and Weeder [43] to determine putative binding sites in the cluster promoters of *F. fujikuroi* and *F. semitectum*. Promoters were defined as 5′ intergenic sequences with a maximum of 1 kb of upstream nucleotides. We compared the occurrence of determined motifs in the cluster to the genome-wide distribution and calculated the significance of motif enrichment using Fisher's exact test [44]. Adjustment for multiple testing was performed using Bonferroni procedure [45]. All determined de-novo motifs were compared to known binding sites in TRANSFAC-database [46].

## Results

### The apicidin F cluster in *F. fujikuroi* consists of 11 genes

A comparison of the *F. fujikuroi* APF gene cluster with the APS cluster in *F. semitectum* revealed a syntenic cluster organization in both fungi: the only differences were the missing *aps10* gene in *F. fujikuroi* and the interchange of *aps2*/*APF2* and *aps3*/*APF3* (Fig. 1A). Beside *APF1* and *APF2* encoding the NRPS key enzyme and the pathway-specific TF, respectively, the cluster in *F. fujikuroi* consists of *APF3*, *APF4*, *APF5*, *APF6*, *APF7*, *APF8*, *APF9*, *APF11* and *APF12*, encoding the following proteins: a putative Δ^1^-pyrroline-5-carboxylate reductase, an aminotransferase, a fatty acid synthase, a *O*-methyltransferase, two cytochrome P450 oxidases, a FAD-dependent monooxygenase, a major facilitator superfamily (MFS) transporter and a cytochrome b5-like reductase, respectively (Table 1).

**Table 1:** Gene name, accession numbers, length and predicted function of the apicidin F cluster and its border gene.

Gene name (accession number)	Length [bp]	Predicted function
***APF1*** (*FFUJ_00003*)	15386	Non-ribosomal peptide synthetase
***APF2*** (*FFUJ_00012*)	1225	bANK transcription factor
***APF3*** (*FFUJ_00013*)	943	Δ^1^-Pyrroline-5-carboxylate reductase
***APF4*** (*FFUJ_00011*)	1412	Aminotransferase
***APF5*** (*FFUJ_00010*)	4995	Fatty acid synthase, alpha subunit
***APF6*** (*FFUJ_00008*)	1150	*O*-Methyltransferase
***APF7*** (*FFUJ_00007*)	1822	Cytochrome P450
***APF8*** (*FFUJ_00006*)	1211	Cytochrome P450
***APF9*** (*FFUJ_00005*)	1918	FAD-dependent monooxygenase
***APF11*** (*FFUJ_00004*)	1920	Major facilitator superfamily transporter
***APF12*** (*FFUJ_00009*)	2885	Cytochrome b5-like
*FFUJ_00014*	2206	Unknown function, probably not belonging to the apicidin F gene cluster

As previously shown, overexpression of the TF-encoding gene *APF2* activated the expression of the otherwise weakly expressed *APF* genes [9]. The elevated expression of the cluster genes enabled us to elucidate a new APS-like SM, APF, which contains four amino acids: *N*-methoxy-l-tryptophan and d-pipecolic acid which are also included in APS, and two different amino acids, l-phenylalanine and l-2-aminooctanedioic acid, which are not incorporated into the APS molecule (Fig. 1B) [10].

**Figure 2:**
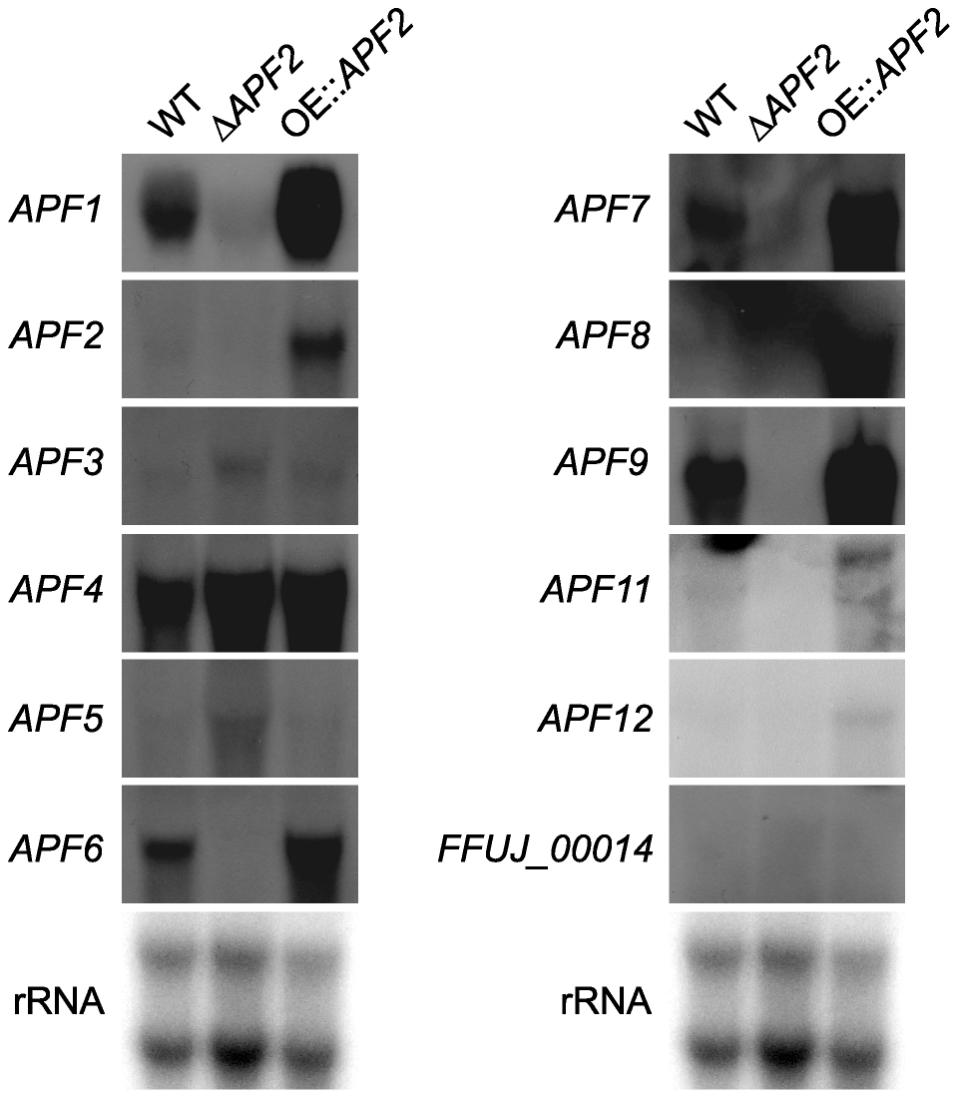
Co-regulation of the apicidin F cluster genes. The WT, Δ*APF2* (TF) and OE::*APF2* were grown for three days in 60 mM glutamine. RNA was isolated from lyophilized mycelia. Northern blot analysis was done as described in methods. As probes, the *APF* genes *1*–*12* and the border gene *FFUJ_00014* were used.

In *F. semitectum*, not all cluster genes were shown to be involved in APS biosynthesis, although they were all controlled by the pathway-specific TF Aps2 [14]. To clearly define the borders of the APF gene cluster and to demonstrate whether all eleven *APF* genes are co-regulated by Apf2 in *F. fujikuroi*, we generated an *APF2* deletion mutant (Δ*APF2*) in addition to the already existing *APF2* over-expressing mutant (OE::*APF2*). The WT, Δ*APF2* and OE::*APF2* strains were grown under nitrogen sufficient (60 mM glutamine) conditions for three days, and the expression of the eleven postulated *APF* genes was monitored by Northern blot analysis. The gene (*FFUJ_00014*) adjacent to the postulated cluster border gene *APF3* was also included to verify our hypothesis that it does not belong to the cluster and is not regulated by Apf2. The expression of most of the genes was indeed affected by deletion and over-expression of *APF2*: *APF1*, *APF6*, *APF7*, *APF8*, and *APF9* were down-regulated in the Δ*APF2* and up-regulated in the OE::*APF2* mutant, while *APF11* and *APF12* transcripts were only detectable in the OE::*APF2* mutant (Fig. 2). *APF3*, *APF4* and *APF5* were not significantly affected in the mutants or even slightly up-regulated in the deletion mutant. As expected, the gene *FFUJ_00014* was not expressed under the given conditions in either strain suggesting that it does not belong to the gene cluster.

### *APF* gene expression depends on nitrogen availability and ambient pH, and expression changes over time

**Figure 3:**
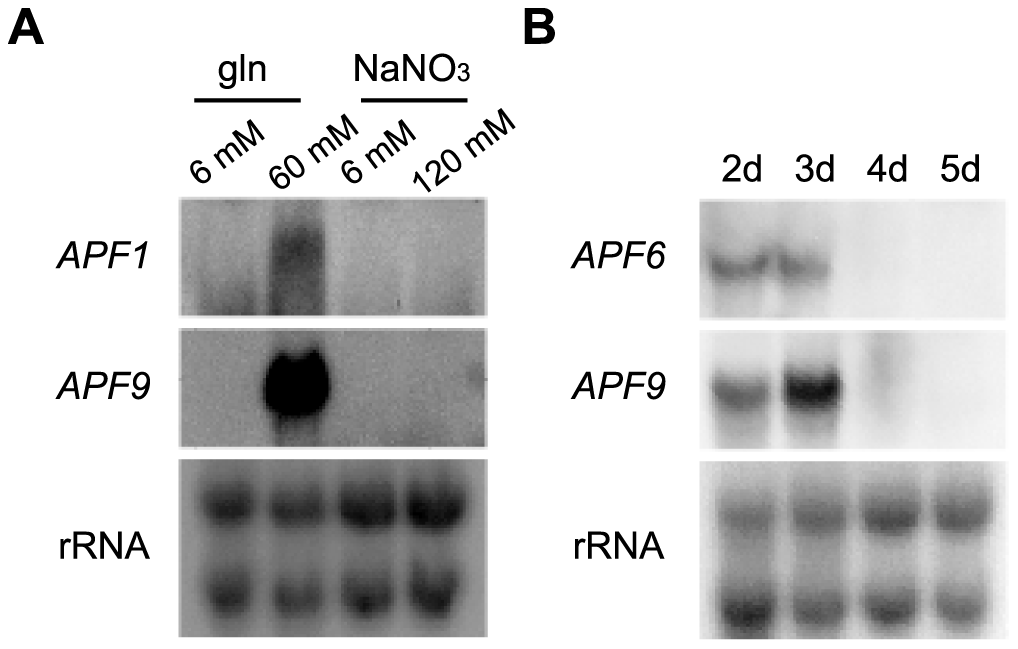
Apicidin F cluster genes are expressed under high amounts of glutamine (gln) from the second to the third day. (A) The WT was grown in four nitrogen conditions, 6 and 60 mM gln and 6 and 120 mM NaNO_3_ for three days. After harvesting, RNA was isolated from the mycelium. *APF1* and *APF9* were used as probes. (B) The WT was grown from the second to the fifth day (d) in 60 mM glutamine. Northern blot analysis was performed with the extracted RNA. *APF6* and *APF9* were used as probes.

Recently we have shown that the expression of most so far characterized SM biosynthetic genes in *F. fujikuroi* is regulated by nitrogen availability and pH [9]. To find the optimal conditions for APF production, the WT was grown for three days in liquid synthetic medium in the presence of low (6 mM) and high (60 mM) amounts of glutamine causing an acidic pH range (5.0–5.5), or 6 and 120 mM NaNO_3_ causing an alkaline pH range (8.0–8.5). Transcripts were detectable only with 60 mM glutamine, indicating that APF cluster genes were induced by saturated amounts of glutamine which confers acidic ambient pH conditions (Fig. 3A). In addition, *APF* genes show a time-dependent expression pattern when grown under optimal production conditions for five days. The transcript levels (represented by *APF6* and *APF9*) reached a peak on the second and third day and were not detectable later on (Fig. 3B).

### The global regulators PacC and AreB, as well as the glutamine synthetase, are involved in the regulation of *APF* gene expression

As the *APF* genes are regulated by nitrogen availability and ambient pH, we studied the impact of the pH-responsive Cys2His2 zinc finger TF PacC [47] and of two nitrogen regulators, the GATA TFs AreA and AreB [22].

**Figure 4:**
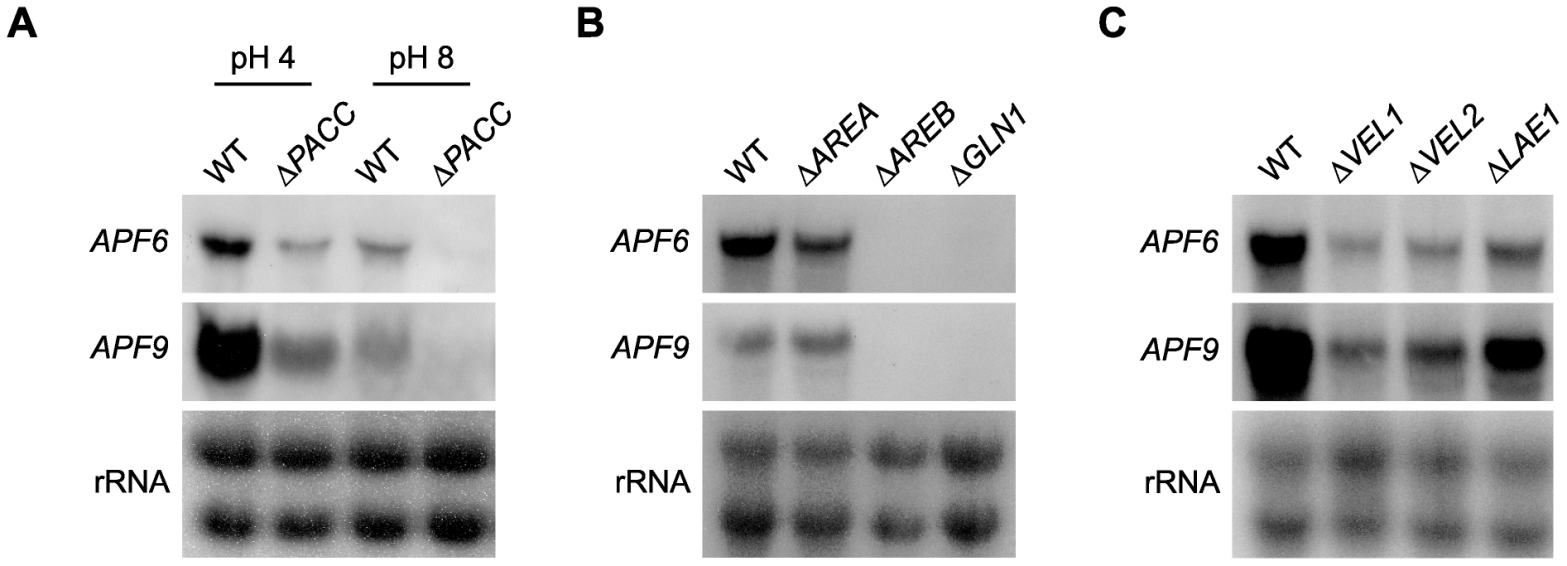
Regulation of the apicidin F cluster. (A) The pH regulator PacC seems to be an activator of the apicidin F genes. The WT and Δ*PACC* were grown for three days under optimal conditions (60 mM glutamine, gln). The cultures were harvested and after washing, the mycelium was shifted into new flasks containing 60 mM gln adjusted to an ambient pH of 4 or 8, respectively. After 2 h the cultures were harvested again. (B) The nitrogen regulators AreB and glutamine synthetase (GS) are activators of the apicidin F gene expression. The WT, Δ*AREA* and Δ*AREB* and the gln auxotroph mutant Δ*GLN1* were grown for three days in 60 mM gln. (C) The WT, Δ*VEL1*, Δ*VEL2* and Δ*LAE1* were grown for three days in 60 mM gln. *APF6* and *APF9* were used as probes for all Northern blot analyses.

To examine the possible impact of PacC on the expression of the *APF* genes, the WT and the Δ*PACC* mutant were grown in 60 mM glutamine. After three days, the mycelium was washed and transferred into flasks with fresh synthetic medium either adjusted to pH 4 or pH 8. Northern blot analysis revealed a significantly higher expression of *APF6* and *APF9* at pH 4 than at pH 8. In the *PACC* deletion mutant, the expression was much lower than in the WT under optimal pH conditions, and undetectable at pH 8, indicating that PacC acts as an activator of the APF biosynthetic genes (Fig. 4A). However, no PacC binding motif (5′-GCCARG-3′) [47] is present in the promoter regions of the *APF* genes suggesting that PacC does not directly bind to these promoters, but rather acts as an indirect activator.

Recent data revealed an involvement of the two GATA TFs AreA and/or AreB in SM gene regulation in *F. fujikuroi* [22], [48]–[50]. In addition, the glutamine synthetase (GS) was shown to be involved in regulation of secondary metabolism in *F. fujikuroi* because the production of the gibberellins, bikaverin and fusarins is abolished in the Δ*GLN1* mutant [23], [29], [51].

To show whether these regulators are also involved in nitrogen regulation of *APF* genes, the WT and the Δ*AREA*, Δ*AREB* and Δ*GLN1* mutants were grown for three days in synthetic medium with 60 mM glutamine. AreA had no impact, neither on the production of APF nor on the expression of *APF* genes. In contrast, no expression of *APF* genes was detected in the Δ*AREB* and Δ*GLN1* mutants (Fig. 4B).

Previously we have shown a strong impact of components of the *velvet* complex (Vel1, Vel2, Lae1) on the expression of genes encoding production of gibberellins, bikaverin, and the mycotoxins fusaric acid and fusarin C [24], [29], [48]. However, the expression of the *APF* genes was only slightly reduced in the Δ*VEL1*, Δ*VEL2* and Δ*LAE1* mutants (Fig. 4C).

### Over-expression of the TF gene *APF2* is able to overcome the nitrogen regulation

Our studies provide evidence that APF production is controlled by a complex regulatory network including several global regulators such as AreB and PacC. In addition, we have previously shown that over-expression of the pathway-specific TF Apf2 led to strong elevation of product yields and allowed structure elucidation of the new compound [9], [10].

**Figure 5:**
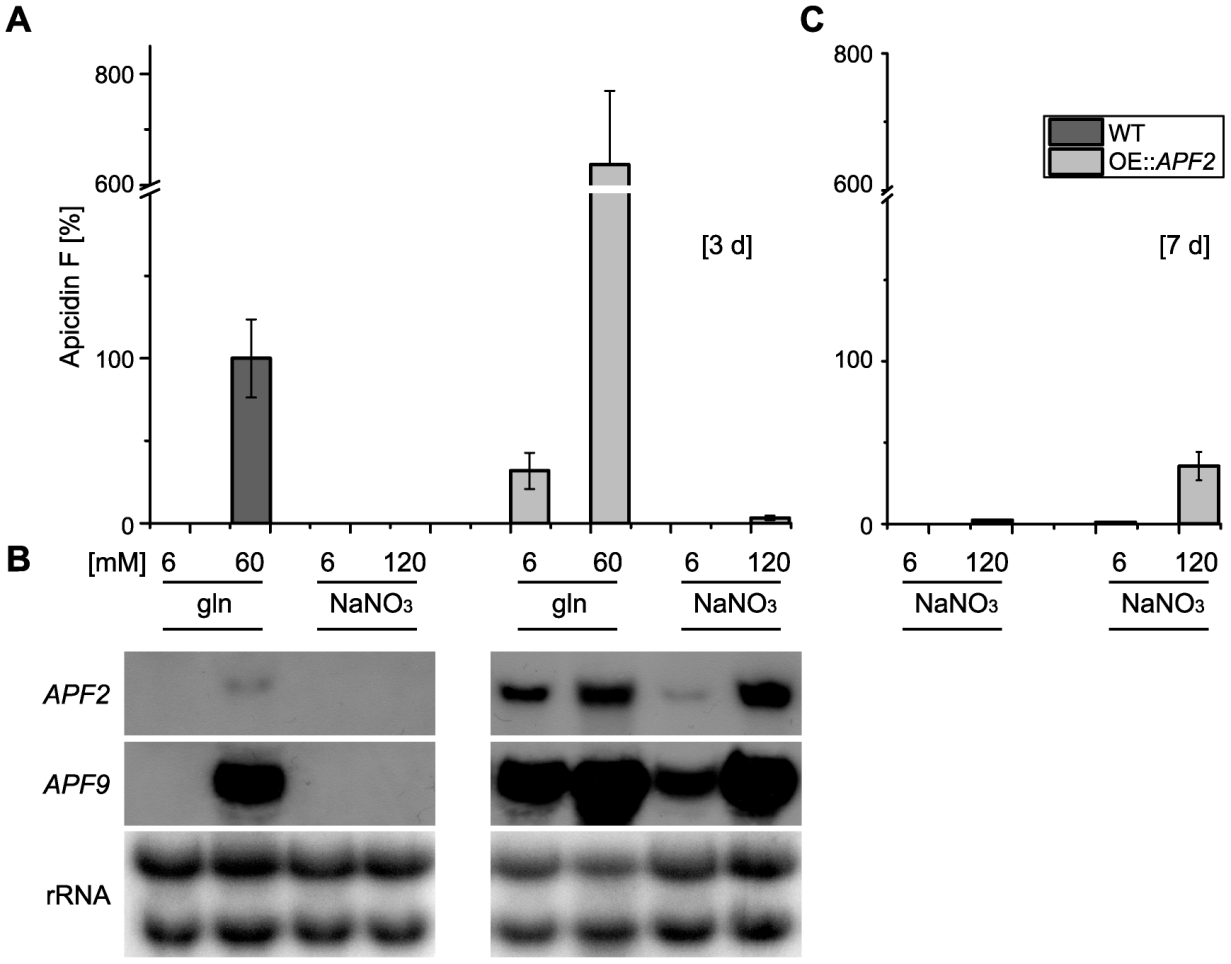
Over-expression of the transcription factor-encoding gene *APF2* (OE::*APF2*) is able to overcome the nitrogen regulation of apicidin F. (A) HPLC-DAD measurement of the extracted mycelium of the WT and the OE::*APF2* mutant after three days. Both strains were grown in four nitrogen conditions, 6 and 60 mM glutamine (gln) and 6 and 120 mM NaNO_3_. Apicidin F was measured at a wavelength of 280 nm. (B) Northern blot analyses of the WT and the OE::*APF2* mutant. Same conditions were used as for the HPLC measurements. *APF2* and *APF9* were taken as probes. (C) HPLC-DAD measurement of the extracted mycelium of the WT and the OE::*APF2* mutant after seven days in 6 and 120 mM NaNO_3_. Product formation was assessed in triplicates and normalized to the WT level.

To determine whether over-expression of *APF2* can overcome repression of *APF* genes under conditions of low nitrogen and at alkaline pH, the WT and the OE::*APF2* mutant were grown under four standard conditions (6 and 60 mM glutamine; 6 and 120 mM NaNO_3_) [9] for three days. APF was analyzed in both the supernatant and the lyophilized mycelium. As expected, the production of APF was significantly increased under optimal production conditions (60 mM glutamine) compared to the WT (Fig. 5A; shown for mycelium extract). However, in contrast to the WT, APF was also produced in media with low amounts of glutamine, and even with high amounts of nitrate causing an alkaline pH (Fig. 5A). The expression of *APF* genes (shown for *APF2* and *APF9*) was increased under all four nitrogen/pH conditions compared to the WT (Fig. 5B). After seven days, production with high NaNO_3_ was increased even further, while only low production was observed in the WT (Fig. 5C). Therefore, the results suggest that the over-expression of the cluster-specific TF gene could partially override the repression of *APF* genes under non-favoring low nitrogen and alkaline pH conditions.

### The pathway-specific TF Apf2 belongs to the class of bANK TFs

**Figure 6:**
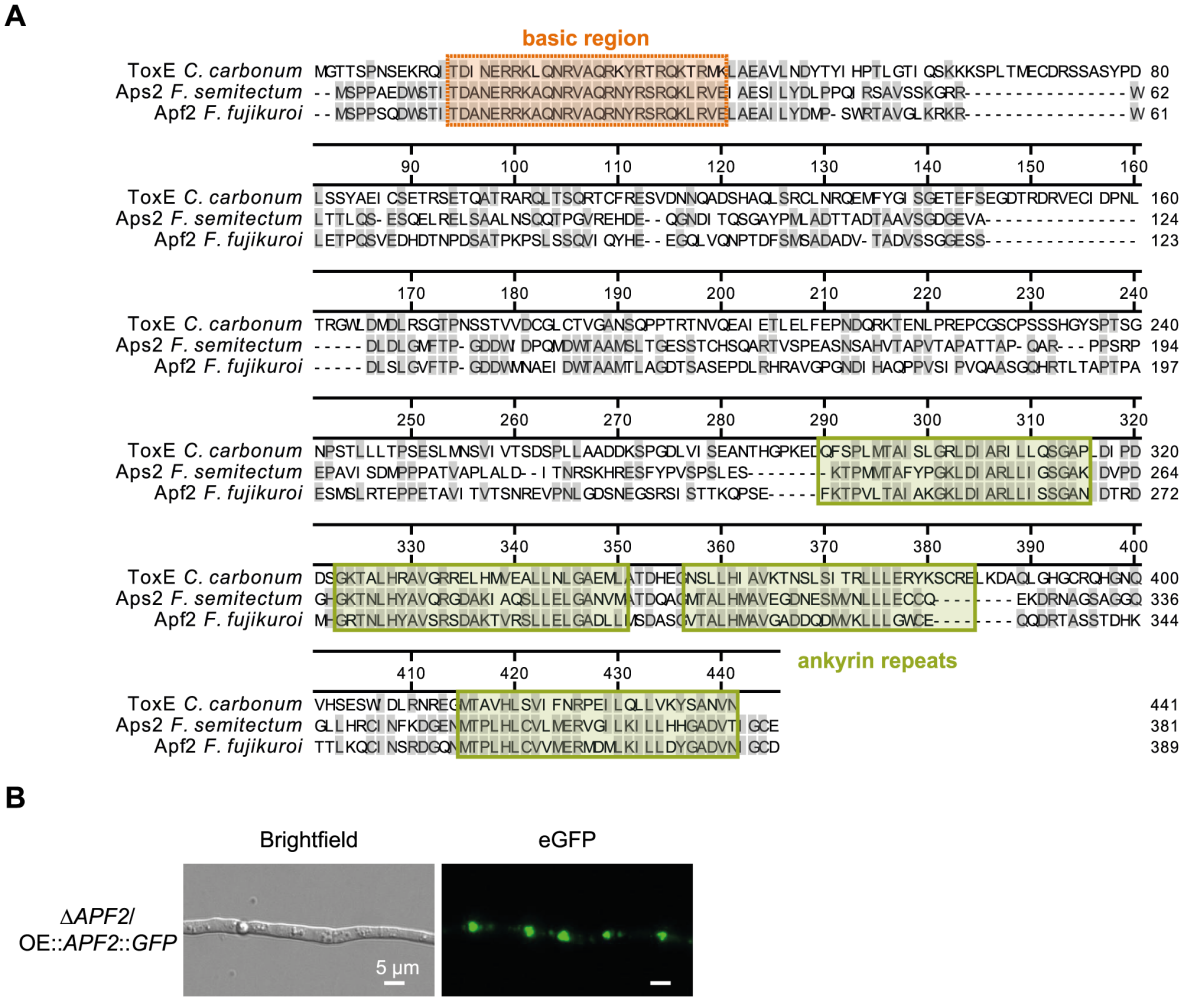
The transcription factor (TF) Apf2 contains a basic DNA binding domain, four ankyrin repeats and is localized in the nucleus. (A) ClustalW alignment with amino acids of *Cochliobolus carbonum* ToxE (AFO38874), *Fusarium semitectum* Aps2 (GQ331953) and *Fusarium fujikuroi* Apf2 (FFUJ_00012). Identical amino acids are highlighted in grey, the positions of the domains are highlighted in either orange (basic DNA binding domain) or green (four ankyrin repeats) and based on [55]. (B) The TF was fused to green fluorescent protein (GFP) at the C-terminus. The Δ*APF2* mutant was used as background. The two strains were grown for one day in 60 mM glutamine. Size of scale bars is indicated. A supplemental figure with controls is depicted in Fig. S2 in File S1.

Most of the so far identified pathway-specific TFs of fungal SM gene clusters belong to the Zn(II)_2_Cys_6_-type fungal-specific transcription factors. In contrast, bioinformatic analysis revealed that Apf2 and Aps2 in *F. fujikuroi* and *F. semitectum*, respectively, contain a basic DNA-binding region at the N-terminus which is usually found in bZIP TFs, but they do not contain the characteristic leucine zipper domain. Instead, four C-terminal ankyrin repeats were identified that were shown to confer protein-protein interaction of regulatory proteins [52]. This unusual class of TFs was called bANK (**b**asic region & **ank**yrin repeats) by Bussink et al. [53]. So far, only one TF of this class has been characterized in more detail: ToxE of the *Cochliobolus carbonum* HC-toxin biosynthetic gene cluster. Similarly to Aps2 and Apf2, ToxE was shown to be essential for the production of a cyclic tetrapeptide, HC-toxin [54]. An amino acid alignment of the three TFs revealed a high level of sequence identity, especially in the functional domains of the proteins with an overall identity of 31% between *Fusarium* Apf2/*C. carbonum* ToxE and 51% between Aps2 and Apf2 proteins (Fig. 6A).

For *C. carbonum*, it was shown that ToxE binds DNA and therefore acts as a transcriptional activator [55]. To show whether Apf2 is indeed localized in the nucleus as assumed, we generated an *APF2*::*GFP* fusion construct and transformed it into the Δ*APF2* background. The ectopic integration of the plasmid was verified by diagnostic PCR (Fig. S1 in File S1). The production of APF was restored in transformants which have integrated the fusion construct, providing evidence that the fusion protein was functional. Epifluorescence microscopy of the mutants then revealed a permanent localization of this TF in the nucleus (Fig. 6B, Fig. S2 in File S1).

### Identification and mutation of the putative DNA-binding motif

After demonstrating that Apf2 acts as transcriptional activator of *APF* gene expression and that it is localized in the nucleus, we wanted to identify its DNA-binding motif in the promoters of the *APF* cluster genes. Due to the high similarity between the APS and APF gene clusters, we assumed a conservation of regulatory elements in the promoters of orthologous genes. Sequence analysis of the promoters of *F. fujikuroi* and *F. semitectum APF*/*APS* genes using the Phylocon algorithm [41] revealed a putative binding motif with the consensus sequence 5′-TGACGTGA-3′ which is significantly enriched in the promoters of *APF* and *APS* cluster genes compared to the genome-wide distribution of the motif in *F. fujikuroi* (P-Value <0.01). This motif can be found in the promoters of all cluster genes except the *APF2* gene itself in *F. fujikuroi*, while it is also not present in the promoter region of *aps3* and *aps10* in *F. semitectum*. In the bidirectional promoters of the genes *APF1*/*APF11*, *APF4*/*APF5* and *APF7*/*APF8*, this motif is present only once. A query against the TRANSFAC database [46] revealed a similar, yet perfectly palindromic sequence, 5′-TGACGTCA-3′, which has been shown to be the recognition site for the mammalian global bZIP TF CREB (**c**AMP-**r**esponse **e**lement-**b**inding protein) that is involved in glucose homeostasis [56].

**Figure 7:**
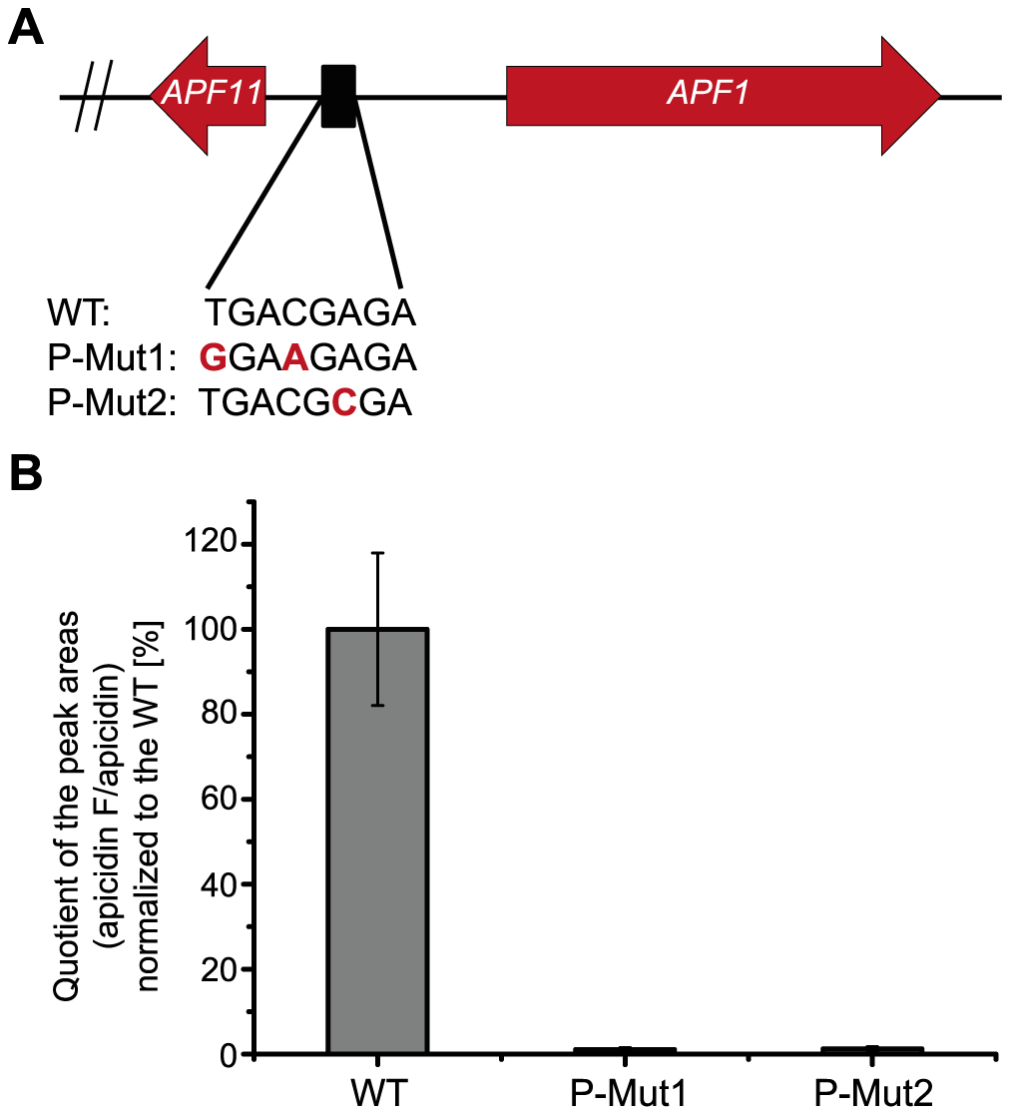
Mutation of the putative “Api-box” motif in the promoter region of *APF1* (NRPS) and *APF11* (transporter) resulted in reduced production of apicidin F. (A) Bioinformatic searches revealed an eight-base-pair motif with the consensus sequence 5′-TGACGTGA-3′ that was found in all promoters of the apicidin F cluster except in the promoter region of the transcription factor (TF)-encoding gene itself. In our study, we created two mutants with point mutations in the *APF1*/*APF11* promoter (P-mut1 and P-mut2, for the strategy see Fig. S3 in File S1). (B) Biosynthesis of apicidin F was monitored with HPLC-HRMS. After growth for three days in 60 mM glutamine, the cultures of the WT and the two mutants P-mut1 and P-mut2 were harvested. Apicidin F was extracted from lyophilized mycelium. 10 µL of a 1 µg/mL apicidin solution (internal standard) were added to 90 µL of the sample. For the calculation, the peak area of apicidin F [M+H]^+^ (646.3235±0.0032) was divided with that of apicidin [M+H]^+^ (624.3756±0.0032). Product formation was normalized to the WT level. Experiment was performed in a triplicate.

In order to gain experimental proof that this motif is indeed a functional cluster-specific TF binding site, probably for Apf2, two different point mutations were introduced in the single motif upstream of *APF1* encoding the NRPS. First, a combination of two nucleotide exchanges was introduced into position 1 and 4 resulting in the motif “P-Mut1” (5′-**G**GA**A**GAGA-3′ instead of 5′-**T**GA**C**GAGA-3′). For the palindromic recognition site of CREB in mammals, the mutation of either of these positions resulted in the greatest impact on corresponding gene expression [57], [58]. In a second approach (“P-Mut2”), position 6 was mutated resulting in a sequence motif of 5′-TGACG**C**GA-3′ instead of 5′-TGACG**A**GA-3′ (Fig. 7A).

Transformants which have integrated the vectors with the indicated promoter mutations into the *APF1* locus were identified by diagnostic PCR (Fig. S3 in File S1). Transformants with correct P-Mut1 and P-Mut2 promoter mutations were grown under APF producing conditions. HPLC-HRMS analysis revealed an almost total loss of APF production for both types of mutations (Fig. 7B). This result supports our suggestion that this motif is the cluster-specific recognition site for a cluster-specific TF, probably Apf2. According to the “Tox-box” described for *C. carbonum* [55], we named this motif “Api-box”.

### Targeted *APF* gene deletion to elucidate the APF biosynthetic pathway

**Figure 8:**
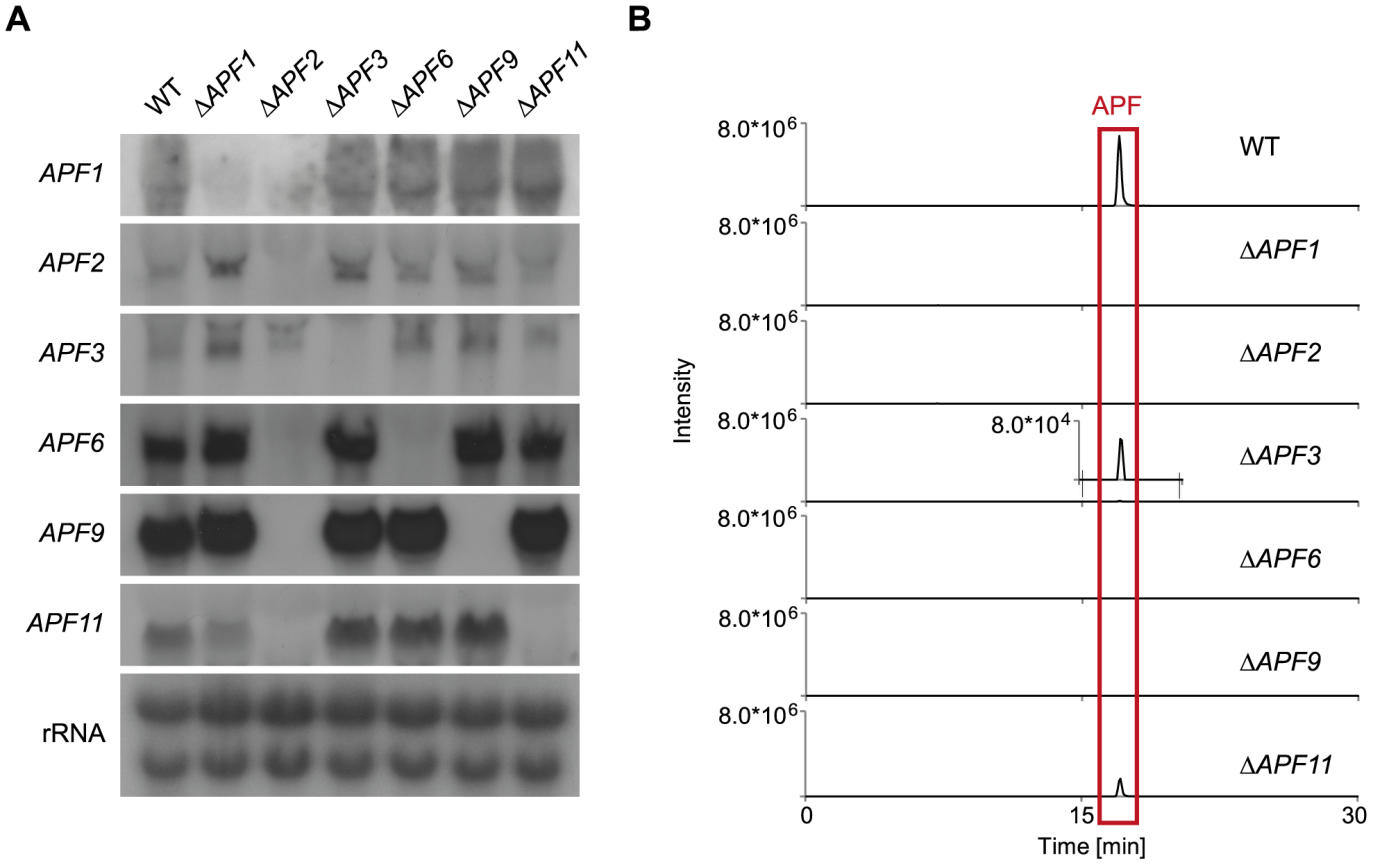
Influence of single *APF* gene deletions on the expression of the remaining genes and the production of apicidin F in these mutants. (A) The single gene deletions have no impact on the expression of the remaining genes. Only deletion of *APF2* (TF) resulted in down-regulation of all genes except of *APF3*. For this experiment, the WT and the single deletion mutants of the apicidin F gene cluster were grown for three days in 60 mM glutamine (gln). After harvesting, a northern blot was performed and hybridized with indicated probes *APF1*, *APF2*, *APF3*, *APF6*, *APF9* and *APF11*. (B) HPLC-HRMS-chromatograms of the culture filtrates of the WT and the single deletion mutants of the *APF* gene cluster grown in ICI with 60 mM gln for three days. Shown are the extracted ion chromatograms for the [M+H]^+^-ion of apicidin F (646.3235±0.0032), the axes are normalized to the WT-level. The deletion mutant of the transporter-encoding gene (Δ*APF11*) still produces WT-levels of apicidin F. Δ*APF3* produces apicidin F as well but in a decreased manner. Analysis of the mycelium extracts led to comparable results (Fig. S10 in File S1).

Our analyses revealed that targeted replacement of *APF1* and *APF2* led to the loss of APF production in these mutants. To gain a deeper insight into the biosynthetic pathway, we deleted those genes that were expected to be involved in the supply of the amino acid precursors for the NRPS. Single gene deletions were generated for *APF3* (pyrroline reductase), *APF6* (*O*-methyltransferase), *APF9* (FAD-dependent monooxygenase) and *APF11* (MFS transporter) gaining at least two independent transformants in each case. Diagnostic PCR and Southern blot analyses (Fig. S4–S9 in File S1) verified the homologous integration of the *HPH* resistance cassette as well as the absence of WT signal. The WT and the deletion mutants (Δ*APF1*, Δ*APF2*, Δ*APF3*, Δ*APF6*, Δ*APF9* and Δ*APF11*) were grown under producing conditions (60 mM glutamine) for three days and analyzed for the expression of the remaining cluster genes (Fig. 8A). Northern blot analysis clearly showed that the deletion of either of these cluster genes did not affect the expression of the remaining genes. Only the deletion of *APF2* showed the expected down-regulation of most of the *APF* genes except for *APF3*. The expression of the *APF* genes independently from the presence of the remaining genes was a good prerequisite for identifying intermediates of the biosynthetic pathway.

**Figure 9:**
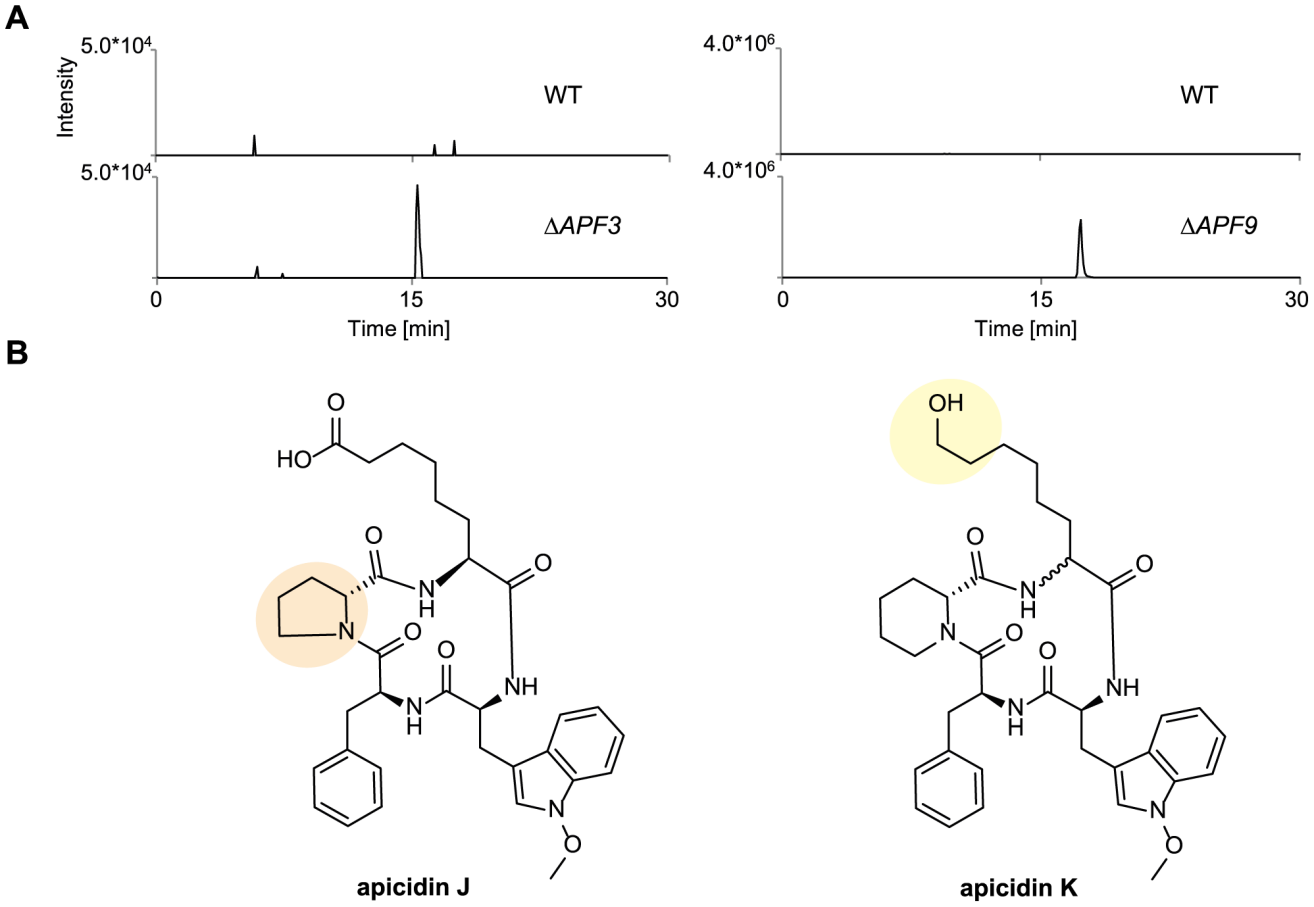
Deletion of the cluster genes *APF3* and *APF9* revealed new analogs of the apicidin F biosynthetic pathway. (A) HPLC-HRMS-chromatograms of the culture filtrates of the WT and the single deletion mutants of *APF3* (Δ*APF3*) and *APF9* (Δ*APF9*) grown in ICI with 60 mM glutamine for three days. Shown are the extracted ion chromatograms for the [M+H]^+^-ion of proline apicidin F (apicidin J, 632.3079±0.0032, left) and for the [M+H]^+^-ion the Δ*APF9*-product (apicidin K, 632.3443±0.0032, right). The axes are normalized to the WT-level. (B) Structures of the two identified products: apicidin J and apicidin K.

Subsequently, the culture filtrate of the WT and the single deletion mutants was analyzed for APF accumulation by use of sensitive HPLC-HRMS. Deletion of *APF6* and *APF9* completely abolished APF production while product formation was reduced 100-fold in both the mycelium and the culture fluid of the mutant Δ*APF3* (Fig. 8A; shown for culture filtrate). Only traces of APF were detected in the mycelium of the Δ*APF6* mutant (Fig. S10 in File S1). In *F. semitectum*, deletion of *aps3* and *aps9* resulted in the formation of APS derivatives due to the remarkable ability of the NRPS Aps1 to incorporate related amino acids. Therefore, the incorporation of proline instead of pipecolic acid was shown for the Δ*aps3* mutant (apicidin B) while 2-amino-8-(S)-hydroxydecanoic acid, a precursor of 2-amino-8-oxodecanoic acid, was utilized upon loss of *aps9* (apicidin D_2_) [14]. For this reason, the *F. fujikuroi* Δ*APF3* and Δ*APF9* mutants were studied in more detail for the appearance of new compounds. HPLC-HRMS analysis revealed new peaks in the extracted mycelia as well as in the culture filtrates of both mutants (Fig. 9A), while no new peak was observed for the Δ*APF6* deletion mutant. Furthermore, Δ*APF11* still produced WT levels of APF in both the supernatant and the mycelium extract (Fig. 8B and Fig. S10 in File S1).

### Structural identification of the biosynthetic analogs produced by the Δ*APF3* and Δ*APF9* mutants

Further characterization of the two biosynthetic analogs in the Δ*APF3* and Δ*APF9* mutants via HPLC-HRMS revealed molecular formulas C_34_H_41_N_5_O_7_ for the Δ*APF3*-product and C_35_H_45_N_5_O_6_ for the Δ*APF9*-product, respectively (Fig. 9A). The molecular formula of the Δ*APF3*-product fits a hypothetical APF-structure with proline incorporated instead of pipecolic acid. This derivative is comparable to apicidin B that was identified in the corresponding Δ*aps3* mutant in *F. semitectum* [14]. This so far unknown Δ*APF3*-product was named apicidin J.

The molecular formula of the Δ*APF9*-product fits a hypothetical APF-structure with 2-amino-8-hydroxyoctanoic acid instead of 2-aminooctanedioic acid. This derivative is comparable to apicidin D_2_ that was identified in the corresponding Δ*aps9* mutant in *F. semitectum* [14]. The new APF derivative found in the Δ*APF9* mutant was designated as apicidin K.

To accumulate sufficient amounts of apicidins J and K for structure elucidation, *APF2* was over-expressed in the Δ*APF3* and Δ*APF9* mutant backgrounds generating Δ*APF3*/OE::*APF2* and Δ*APF9*/OE::*APF2* double mutants. This approach resulted in a ca. 10-fold enhanced production of the derivatives. The structures were elucidated using a method already applied for the structural elucidation of APF [10]. As the amounts of apicidin J were insufficient for NMR-spectra, a combination of hydrolysis, Marfey's derivatization and partial hydrolysis, followed by HPLC-HRMS/MS analysis, was used (Fig. S11 in File S1, Fig. S12 in File S1). These methods confirmed the incorporation of proline instead of pipecolic acid by Apf1 in the Δ*APF3* mutant. The product apicidin J consists of *N*-methoxy-l-tryptophan, l-phenylalanine, d-proline and l-2-aminooctanedioic acid (Fig. 9B) and is comparable to apicidin B in *F. semitectum* [14].

Based on 1D- and 2D-NMR experiments, the amino acid composition as well as their sequence could be determined for apicidin K, the product of the Δ*APF9* mutant. This analysis revealed a modified side chain containing a terminal hydroxyl group in the structure of apicidin K (Table S2, Fig. S13–16 in File S1).

To elucidate the stereochemistry of three of the four amino acids (*N*-methoxy-l-tryptophan, l-phenylalanine, d-pipecolic acid), hydrolysis and Marfey's derivatization were performed (Fig. S17 in File S1). The stereochemistry of the fourth amino acid, 2-amino-8-hydroxyoctanoic acid, could not be determined due to missing reference compounds (Fig. 9B). However, based on structural similarity of apicidin K with the corresponding analog apicidin D_2_ in the *F. semitectum* Δ*aps9* mutant [14], it can be assumed that the stereochemistry is the same as for l-2-aminooctanedioic acid in APF. The only difference is that apicidin K is hydroxylated at C-8 (Fig. 9B). The stereochemistry of the amino acid should not be affected by this modification. Nevertheless, there is no complete proof for the stereochemistry.

In *F. fujikuroi*, both apicidin J and apicidin K were only produced in the corresponding deletion mutants, but not in the WT. In contrast, apicidin D_2_ and B co-occurred with APS in the *F. semitectum* WT, though in comparatively low amounts [14].

Finally, to study the role of the two putative P450 monooxygenases, Apf7 and Apf8, an existing **c**ytochrome **P**450 **r**eductase deletion mutant (Δ*CPR*) was analyzed for APF production. P450 monooxygenases depend on the electron transfer from a reduction equivalent to the P450 monooxygenase catalyzed by the NADPH-dependent Cpr. Therefore, the activity of P450 monooxygenases, e.g. Apf7 and Apf8, is inhibited in the Δ*CPR* mutant due to the missing electron donor protein. Subsequently, the Δ*CPR* mutant is useful to study the general involvement of Apf7 and/or Apf8 in APF biosynthesis. The Δ*CPR* mutant was grown under optimal APF production conditions for three days, and the culture supernatant was analyzed via HPLC-HRMS. APF production was completely abolished in the Δ*CPR* mutant and no additional APF derivatives were detected, underlining the essential function of Apf7 and/or Apf8 in biosynthesis (data not shown).

### Cytotoxic effects of APS and APF on Hep G2-cells

**Figure 10:**
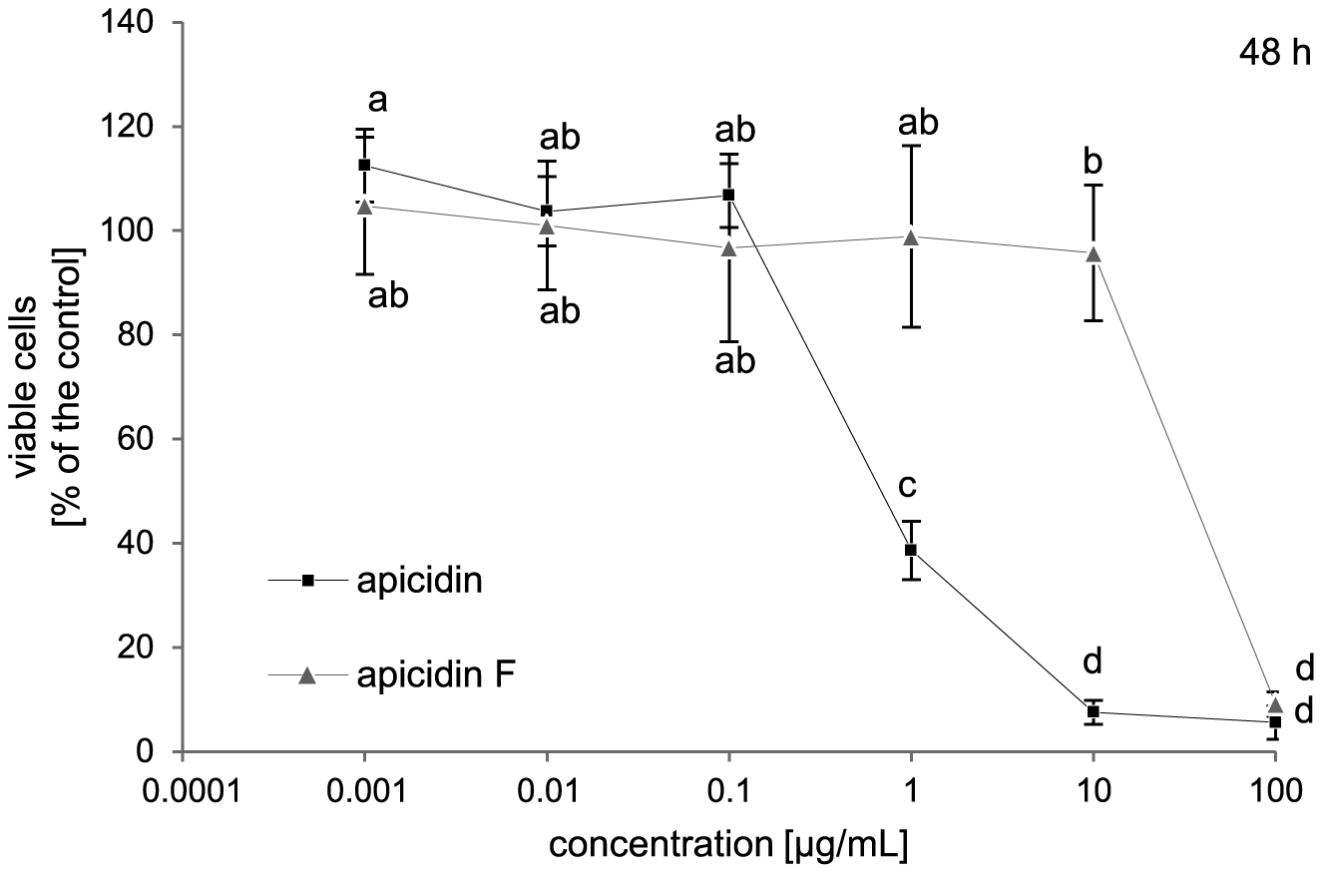
Cytotoxicity of apicidin F and apicidin. Hep G2 cells were incubated with concentrations from 0.001 µg/mL to 100 µg/mL apicidn F or apicidin, respectively for 48 h. Cytotoxicity was determined using the CCK-8 assay. The values of the samples are shown in comparison to the solvent treated negative control (100%). Values are means ± S.D. (n = 9 samples). The letters a-d indicate four groups of samples which differ significantly from the samples labeled with a different letter according to the ANOVA with the Tukey post hoc test (p≤0.05).

Due to the elevated product yields in the OE::*APF2* mutant strain, we were able to compare the biological activity of APS with that of APF. Both compounds were applied to Hep G2 cells and the cytotoxic effects were evaluated using the CCK-8-assay that determines the number of viable cells via the activity of dehydrogenases [40] (Fig. 10). The data clearly showed a significantly lower cytotoxic effect for APF than for APS in both assays. The IC_50_ values calculated from the curves represent this contrasting effect. APS had an IC_50_ value of 1.3 µM whereas the IC_50_ value of APF was 110 µM, about a factor of 100 higher.

## Discussion

Recent genome sequencing of the rice pathogen *F. fujikuroi* provided new insight into the genetic capacity to produce a wide collection of natural compounds. However, transcriptome and HPLC-HRMS-based metabolome analyses indicated that most of the 45 putative gene clusters are cryptic and not expressed under standard laboratory conditions. Several approaches have been shown to activate dormant fungal gene clusters, e.g. by manipulating the activity of global and gene cluster-specific regulators, or by altering the chromatin landscape by either genetic or chemical manipulation of chromatin modifiers [1], [59].

In the present work, we focused on molecular characterization and regulation of the APF gene cluster in *F. fujikuroi* by use of a combination of genetic approaches (gene deletions, over-expression, promoter mutations) and chemical analyses of APF and APF-like derivatives produced by the WT and mutant strains.

### The phylogenetic origin of the APF gene cluster

Comparison of the *F. fujikuroi* genome with those of the six other sequenced species in the genus revealed that only a small number of gene clusters are conserved among these species. Two out of the 45 identified gene clusters, one that includes a polyketide synthase gene (PKS19) and another that includes a NRPS gene (NRPS31), are unique to *F. fujikuroi* and not present in any other sequenced *Fusarium* genome [9]. Phylogenetic analysis of all so far characterized fungal NRPSs revealed the highest similarity (66% identity) of NRPS31 with Aps1 of the distantly related species *F. semitectum* that was shown to be the key enzyme of APS biosynthesis [14]. Beside the sequence similarities between the two NRPSs, the adjacent genes are highly syntenic in both fungi, suggesting that *F. fujikuroi* produces an apicidin-like SM.

There are only a few reports of the biosynthesis of cyclic tetrapeptides in fungi. The maize pathogen *C. carbonum* produces HC-toxin, a cyclic tetrapeptide with the structure d-Pro-l-Ala-d-Ala-l-Aeo, where Aeo stands for 2-amino-9,10-epoxi-8-oxodecanoic acid [60]. Recently, genome sequencing of *Alternaria jesenskae* revealed orthologs of each of the known seven *TOX2* genes from *C. carbonum*, and the production of HC-toxin was discovered in this fungus [61]. APS and APF production by *F. semitectum* and *F. fujikuroi*, respectively, and production of trapoxin by the fungus *Helicoma ambiens* are three more examples for fungal cyclic tetrapeptides [10], [12], [62]. All of them show HDAC inhibitor activities and contain similar amino acids, such as Aeo or Aeo derivatives. Comparison of the gene clusters responsible for APS, APF and HC-toxin biosynthesis showed that the closest known homologs of many of the *APS*/*APF* genes are the genes of the HC-toxin cluster in *C. carbonum*. Another common feature of these gene clusters is the presence of a rather atypical bANK-type TF (ToxE and Aps2/Apf2) containing both a basic DNA-binding domain and four ankyrin repeats. ToxE/Aps2/Apf2-like TFs with reasonable amino acid identity and structure were recently also found in the genomes of *Pyrenophora tritici-repentis*, *Setosophaeria turcica* and *Glomerella cingulata*, and in all cases the TF-encoding genes are adjacent to four-module NRPS genes [61]. The DNA binding motifs for ToxE in *C. carbonum* (5′-ATCTCNCGNA-3′; [55]) and Apf2 in *F. fujikuroi* (5′-TGACGTGA-3′; this work) differ despite the high sequence similarity in the basic DNA binding and ankyrin domains of both TFs.

These gene clusters might have a common phylogenetic origin because of the similar cluster organization, the presence of an atypical bANK-type TF, and the fact that fungal species in distantly related genera contain similar gene clusters and produce structurally related cyclic tetrapeptides. Furthermore, the position of the gene cluster in *F. fujikuroi* at the far end of chromosome I may support our hypothesis that this cluster was acquired by horizontal gene transfer (HGT) from an unknown organism. Genomic regions close to chromosome ends are more prone to recombination than telomere-distal regions and genes or gene clusters acquired by HGT are often found in sub-telomeric positions in fungi [63]. Furthermore, none of the so far sequenced related *Fusarium* species from the *Gibberella fujikuroi* species complex, e.g. *F. verticillioides*, *F. mangiferae* or *F. circinatum*, contain this gene cluster, or parts of it, whereas most of the other SM gene clusters are present and highly syntenic in the genomes of these *Fusarium* species [9]. Another possibility would be that an ancestor of the genus *Fusarium* had a similar gene cluster in the genome which was lost during evolution in most of the *Fusarium* species except for *F. semitectum* and *F. fujikuroi*. The sequence differences between the cluster genes in the latter two species, especially in the adenylation domains of both NRPSs, probably led to the differing substrate specificities of both key enzymes. As a consequence, two of the four incorporated amino acids vary between APF and APS [10].

### APF shows lower cytotoxicity on Hep G2 cells compared to APS

Our previous *in vitro* studies of the antimalarial activity of APF against *P. falciparum* revealed a three-fold lower activity compared to apicidin (IC_50_-value of 0.67 µM) [10]. In this study we demonstrated an even lower (100-fold) cytotoxic effect of APF on Hep G2 cells compared to apicidin. These data show that the exchange of amino acids in structurally related cyclic tetrapeptides leads to altered biological activities. Structure-activity relationship studies are an efficient tool to develop new effective drugs, also in the case of HDAC inhibitors. By using synthetically modified apicidins a strong impact of the amino acid composition on HDAC inhibitory activity has been demonstrated [18]–[20]. For example, an exchange of Aoda for 2-aminooctanedioic acid (modified APS) led to a 200-fold decrease in activity against *P. falciparum* and an about 15-fold decrease in inhibition of HDAC activity in human HeLa cells [19]. In contrast, the additional phenylalanine in APF instead of isoleucine probably overrides the decrease in activity against *P. falciparum*, but amplifies the lower effect on mammalian cells.

### The APF biosynthetic pathway

To gain a better insight into the biosynthesis of APF, several cluster genes were deleted and the generated knock-out mutants were analyzed for their ability to produce APF or related compounds by HPLC-HRMS. The structure elucidation was carried out using HPLC-HRMS and/or NMR. and NMR.

Previously, we have shown that the *F. fujikuroi* Apf1 incorporates four amino acids: *N*-methoxy-l-tryptophan, l-phenylalanine, d-pipecolic acid (d-pip) and l-2-amino-8-octanedioic acid (l-Aoc) [10] which have to be activated by the four adenylation domains of the NRPS. While the proteinogenic amino acid l-phenylalanine can be directly activated, the non-proteinogenic amino acids have to be synthesized via product-specific enzymes, probably encoded by cluster genes. One of them is d-pip which occurs in many natural cyclic tetrapeptides. First, l-pip has to be built by the activity of Δ^1^-pyrroline-5-carboxylic acid (P5C) reductase (Apf3) from Δ^1^-pyrroline-6-carboxylic acid (P6C) which might derive from l-lysine [64]. For epimerization of l-pip to d-pip a racemase gene or an epimerase domain of the NRPS is needed. The HC-toxin cluster contains an alanine-racemase that is capable of converting l-alanine into d-alanine [65]. However, the APF cluster does not contain any racemase-encoding gene. Another possibility is that the NRPS itself comprises an epimerization domain as shown for the NRPS HTS1 [(S/A)RTXGWFT(T/S)] which enables the conversion of l-proline to d-proline [65]. A similar motif (SRTVGWFTT) was found in the sequence of Apf1 suggesting that Apf1 is able to epimerize l-pip to d-pip.

To experimentally determine the postulated function of Apf3 as P5C reductase that provides l-pip, we analyzed Δ*APF3* mutant for APF production. The main product of this mutant is apicidin J that contains d-proline instead of d-pip, confirming the involvement of Apf3 in l-pip formation (Fig. 9). The same incorporation of d-proline was demonstrated for the *aps3* deletion mutant in *F. semitectum* [14] indicating a low substrate specificity of the corresponding amino acid activation domain of Aps1/Apf1. However, small amounts (about 100-fold less) of APF were still detectable in the mutant by HPLC-HRMS analysis (Fig. 8B, 9A). This is not surprising because *F. fujikuroi* has two additional putative P5C reductases (FFUJ_09318 and FFUJ_03207) having 28% and 23% identity to Apf3, respectively. P5C reductases are involved in the last step of l-proline biosynthesis in almost all organisms catalyzing the conversion from P5C to l-proline [66]. *In vitro* experiments with recombinant P5C reductase from *E. coli* showed that this enzyme also catalyzes the conversion of P6C to l-pip [67]. We propose that these remaining reductases produce l-proline as usual and perhaps also small amounts of l-pip. Both precursors can be incorporated by the NRPS Apf1 to synthesize either apicidin J or APF, respectively.

The modification of l-tryptophan to *N*-methoxy-l-tryptophan was proposed to be catalyzed only by one enzyme, the putative *O*-methyltransferase Aps6 [14]. However, as the nitrogen atom in l-tryptophan has to be first oxidized before it can be methylated by an *O*-methyltransferase, we suggest that there are probably two enzymes needed. There is only one example known in which the methoxylation of a nitrogen atom can be catalyzed by a single enzyme: the cercosporin biosynthesis in *Cercospora nicotianae*. However, in this case, the responsible enzyme contains both a putative *O*-methyltransferase domain at the N-terminus and a putative FAD-dependent monooxygenase domain at the C-terminus [68]. Apf6, however, has only an *O*-methyltransferase domain. Therefore, we suggest that the oxidation of the nitrogen to form *N*-hydroxy-l-tryptophan could be catalyzed by one of the two P450 monooxygenases, Apf7 or Apf8. In *Beauveria bassiana* it has been shown that a P450 monooxygenase catalyzes the selective *N*-hydroxylation of 2-pyridone in the tenellin biosynthetic pathway [69]. It is noteworthy that deletion of *aps7* in *F. semitectum* led to the production of apicidin E that lacks the keto group at the Aoda residue indicating that this P450 is responsible for the hydroxylation of the aliphatic chain [14]. Accordingly, we suggest that the other P450, Apf8, might be responsible for the oxidation of l-tryptophan.

In a second step, Apf6 is predicted to convert *N*-hydroxy-l-tryptophan to *N*-methoxy-l-tryptophan. As expected, the Δ*APF6* mutant is not able to produce APF, or any alternative product, suggesting that that Apf1 does not recognize *N*-hydroxy-l-tryptophan or l-tryptophan as a substrate. Similarly, deletion of *aps6* in *F. semitectum* also resulted in the loss of apicidin production, and no derivatives were observed [14].

**Figure 11:**
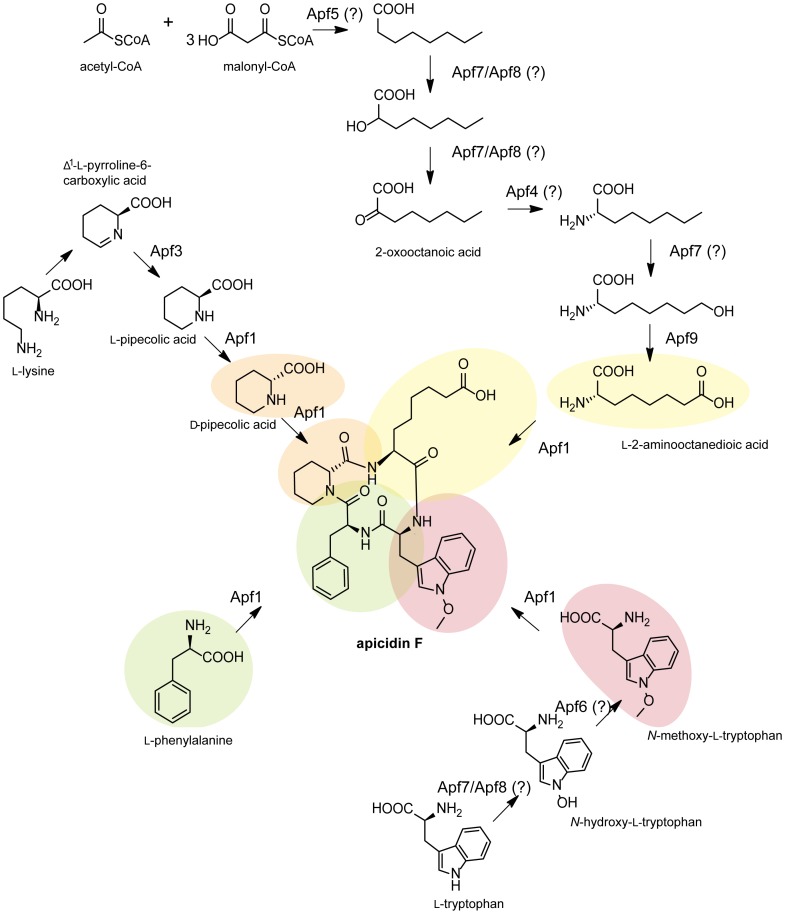
Proposed biosynthetic pathway of apicidin F. Based on our data, we postulated a biosynthetic pathway for apicidin F. Noteworthy, catalyzation steps which are marked with a “?” have not been confirmed experimentally. The NRPS as key enzyme incorporates four different amino acids to produce apicidin F. However, most of the amino acids are non-proteinogenic and therefore, have to be modified by other enzymes of the cluster. l-Phenylalanine is the only proteinogenic amino acid and directly used by Apf1. l-Pipecolic acid is the second precursor of Apf1 and is epimerized to D-pipecolic acid, probably by Apf1 activity, prior to incorporation. It is proposed that lysine is converted to Δ^1^-pyrroline-5-carboxylate (P5C). A P5C reductase catalyzes the transformation to proline. This enzyme could also convert Δ^1^-pyrroline-6-carboxylate (P6C) to l-pip [14]. In our studies, we could demonstrate that deletion of the P5C reductase-encoding gene *APF3* led to the incorporation of proline instead of pip resulting in the production of apicidin J. We claim, therefore, that this enzyme is responsible for the conversion of P6C into l-pip. The NRPS itself has an epimerization domain for the epimerization of l-pip into d-pip. Furthermore, Apf1 incorporates *N*-methoxy-l-tryptophan. We suggest that one of the P450 oxidases (Apf7 or Apf8) *N*-oxidizes l-tryptophan and then the *O*-methyltransferase Apf6 is able to catalyze the methylation of the hydroxy group. The fourth amino acid that is incorporated by Apf1 is l-2-aminooctanedioic acid. It is predicted that the fatty acid synthase-encoding gene *APF5* is involved in the synthesis of the octanoic acid backbone by fixing one acetyl-CoA unit and three malonyl-CoA units [14]. Then one of the P450 oxidases may oxidize this backbone to 2-oxooctanoic acid. The aminotransferase Apf4 is predicted to catalyze the exchange of the keto group with an amino group. The next step would be the oxidation of 2-aminooctanoic acid by one of the P450 oxidases (Apf7 or Apf8). For *F. semitectum*, it could be shown that deletion of *aps7* led to the production of apicidin E (lacks the keto group in comparison to apicidin) [14]. We suggest that the last step is the oxidation of 2-amino-8-hydroxyoctanoic acid to 2-aminooctanedioic acid by the FAD-dependent monooxygenase Apf9 because deletion of the corresponding gene led to the production of apicidin K (lacks the acid group and has a hydroxyl group instead).

The last precursor for APF biosynthesis is the short-chain fatty acid l-Aoc [10]. Most of the remaining cluster genes are potentially involved in formation of this compound. Like the HC-toxin cluster, the *F. semitectum* and *F. fujikuroi* clusters contain a gene that encodes an α-subunit of a fatty acid synthase [9], [14], [70]. This gene (*APF5*) is probably responsible for the condensation of the octanoic acid backbone by successive attachment of three malonyl-CoA units to a single primer molecule of acetyl-CoA. Then, one of the P450 oxidases, probably Apf7, may oxidize octanoic acid to 2-oxooctanoic acid, and finally the putative branched-chain amino acid transaminase Apf4 (homolog of ToxF; [71]) could catalyze the exchange of the keto group of 2-oxooctanoic acid with an amino group. Similar gene functions were predicted for the biosynthesis of Aoda in *F. semitectum*. Because the deletion of *aps7* in *F. semitectum* resulted in production of apicidin E [14], it is very likely that this P450 monooxygenase oxidizes 2-aminooctanoic acid also in *F. fujikuroi*. After this step, the putative FAD-dependent monooxygenase Apf9 is probably involved in conversion of 2-amino-8-hydroxyoctanoic acid into 2-aminooctanedioic acid (Fig. 11). Our analysis of the *APF9* deletion mutant revealed apicidin K, a derivative of APF biosynthesis that contains 2-amino-8-hydroxyoctanoic acid and corresponds to the product of Δ*aps9* in *F. semitectum*, apicidin D_2_ [14]. These data indicate that Apf1 recognizes both, 2-amino-8-hydroxyoctanoic acid as well as 2-aminooctanedioic acid as substrates.

To confirm the essential role of the two P450 oxidases Apf7 and Apf8 in *F. fujikuroi*, we analyzed the deletion mutant of the NADPH-cytochrome P450 reductase gene (Δ*CPR*) [26]. In this mutant, the activity of all P450 monooxygenases, e.g. those of the gibberellin pathway, is inhibited due to the loss of the CPR-catalyzed electron transfer from NADPH to the P450 monooxygenases [26]. Our analysis revealed that APF production is abolished in this mutant (data not shown), demonstrating the essential role of the two P450s in the biosynthesis of APF.

All eleven genes of the cluster were shown to be co-regulated under high nitrogen conditions suggesting that they might be involved in APF biosynthesis. However, our previous studies on the fusarin C gene cluster revealed that only four of the nine co-regulated genes are necessary for the biosynthesis of fusarins [29]. Similarly, not all of the co-regulated genes of the aspyridone gene cluster in *A. nidulans* are involved in the biosynthetic pathway [72]. Likewise, some steps of a SM biosynthetic pathway might be catalyzed by genes which are located outside of the core gene cluster as shown for trichothecene biosynthesis in several *Fusarium* species and the aspyridone gene cluster in *A. nidulans* [72], [73]. Based on our experimental data and by analogy to the pathways for the two closely related cyclic tetrapeptides, APS [14] and HC-toxin [65], we provide a model of the APF biosynthetic pathway in *F. fujikuroi* (Fig. 11).

### The regulation of APF biosynthesis

The regulation of secondary metabolism occurs at pathway-specific and more general levels. Global regulation is achieved by TFs which are encoded by genes that do not belong to any cluster and which may regulate a number of genes that are not involved in secondary metabolism [1]. In addition, SM gene clusters can be regulated by pathway-specific transcription factors, such as Apf2 (see below).

In this work, we studied the impact of global and pathway-specific regulators on APF biosynthesis in *F. fujikuroi*. Beside the role of the specific TF Aps2 in *F. semitectum* [14] and Apf2 in *F. fujikuroi* [9] nothing was known about environmental and genetic factors that can influence the production of APS and APF, respectively. We showed that APF gene expression is induced by high nitrogen concentrations as shown before for fusarin C and fusaric acid biosynthesis [29], [48]. This strong dependency on nitrogen availability suggests the involvement of one or both GATA-type nitrogen regulators, AreA and AreB [22], [49]. The gibberellins were the first SMs whose gene expression and biosynthesis were shown to be induced by nitrogen limitation in an AreA-dependent manner [50]. Recently, we have shown that the second GATA TF AreB is also essential for expression of gibberellin biosynthetic genes in *F. fujikuroi* [22]. While AreA was not responsible for regulating the expression of any known nitrogen-induced SM, AreB was shown to act as positive regulator of nitrogen-induced fusaric acid biosynthetic genes [48]. The APF gene cluster is the second example of a nitrogen-induced SM which is strongly regulated by AreB, but not by AreA. The AreB-dependent gene expression of both gene clusters could be confirmed by a recent microarray analysis comparing the expression profile of the WT with that of the *AREB* deletion mutant (A. Pfannmüller et al., unpublished). In accordance with this finding, there are several putative GATA sequence motifs in the promoters of the APF cluster genes. In addition to AreB, a functional GS protein is essential not only for gibberellin, bikaverin, and fusarin C biosynthesis [48], but also for the expression of *APF* genes and APF production. Insufficient metabolization by the Δ*GLN1* mutant of glucose and, as a consequence, the shortage of ATP, probably causes the down-regulation of secondary metabolism [51].

In addition, *APF* gene expression depends on acidic ambient pH and the presence of the pH regulator PacC. A PacC-dependent regulation has been previously shown for the bikaverin biosynthetic genes whose expression is also induced under acidic conditions, and repressed under neutral or alkaline conditions. Deletion of *PACC* or mutation of the PacC DNA-binding motif in the *BIK1* promoter led to de-repression under repressing alkaline conditions and even higher expression under optimal acidic conditions, indicating that PacC acts as a repressor which seems to directly bind the promoters of *BIK* genes [25]. In contrast, PacC acts as an activator of *APF* gene expression. As we could not identify any PacC binding site in the promoter regions of *APF* genes, PacC seems to act as an indirect activator of the APF gene cluster.

Interestingly, the fungal-specific *velvet* complex seems to play only a minor role for *APF* gene regulation. In contrast, we have previously shown that Vel1, Vel2 and the putative histone methyltransferase Lae1 significantly affect the expression of gibberellin, fusaric acid and fusarin C biosynthetic genes [24], [29], [48].

### The atypical TF Apf2 is essential for biosynthesis of apicidin F

On the basis of available fungal genome sequences, about 60% of fungal SM gene clusters contain a putative regulatory gene. Most of the potential regulators in fungal PKS-encoding gene clusters belong to the Zn(II)_2_Cys_6_-type fungal-specific TFs, whereas NRPS-regulating TFs seem to be more diverse [1].

Over-expression of cluster-specific TFs using strong or inducible promoters was shown to be a sufficient approach to activate normally silent gene clusters and to identify new SMs, e.g. the polyketide asperfuranone [74], the hybrid PKS–NRPS aspyridone [75] and the diterpene compound *ent*-pimara-8(14),15-diene [76] in *A. nidulans* as well as APF in *F. fujikuroi* [9], [10].

Aps2 and Apf2 together with ToxE of the HC-toxin cluster in the plant pathogen *C. carbonum* belong to an atypical class of TFs which combine a basic DNA-binding domain at their N-terminus and four ankyrin repeats at their C-terminus, but do not contain discernible leucine zipper or helix-loop-helix motifs in contrast to typical bZIP TFs ([54]; this study). For ToxE it has been shown that both the basic region and the ankyrin repeats are involved in DNA binding [55]. Due to this unusual domain structure, these TFs were named **b**asic DNA-binding domain-type TF with **ank**yrin-like repeats (bANK proteins) [53]. We showed that *APF2* localizes to the nucleus and that its over-expression positively affects expression of eight out of the eleven cluster genes. Bioinformatic analysis of the promoter sequences of the APF genes revealed an almost palindromic eight-base-pair-motif: 5′-TGACGTGA-3′ which we called “Api-box” in analogy to the “Tox-box” in the HC-toxin cluster in *C. carbonum* [55]. The “Api-box” is present in the promoters of all cluster genes, except Apf2 itself. This motif is conserved among the *F. fujikuroi* and *F. semitectum* APS/APF gene clusters. Mutation of this sequence motif led to a 100-fold decrease of APF production. Although this approach is not direct proof for the interaction of Apf2 with the “Api-box” sequence, it is a strong indication that this motif is essential for *APF* gene activation by binding the pathway-specific TF.

Interestingly, this sequence element is present only in one copy within the bidirectional promoters between *APF1/APF11*, *APF4*/*APF5* and *APF7/APF8*. For the “Tox-box” it has been shown that it is functional in yeast in both orientations [55]. This finding is biologically significant because the “Tox-box” is present only once in the promoters of divergently transcribed genes similarly to the situation of divergently transcribed *APF* genes in *F. fujikuroi*. To confirm without any reservation that the “Api-box” is indeed the binding sequence of Apf2, we will perform yeast one-hybrid assays in the near future.

In this study, we report the identification and functional analysis of the gene cluster responsible for APF biosynthesis in *F. fujikuroi*. The cluster consists of eleven genes while only five of them were shown to be directly involved in APF biosynthesis. Targeted gene replacement of cluster genes and subsequent HPLC-HRMS and NMR analysis revealed two new APF derivatives, apicidin J and K. Based on our data, we provide a model for the APF biosynthetic pathway. Furthermore, we show that the expression of *APF* cluster genes depends on global regulators, such as AreB and PacC, as well as the pathway-specific TF Apf2. Over-expression of this unusual TF gene resulted in a nearly 10-fold elevated product formation under optimal culture conditions. Point mutations in a conserved sequence motif in the promoter of the key gene *APF1* (NRPS) resulted in loss of APF production.

## Supporting Information

File S1 **Supporting figures and tables.** Figure S1. Verification of genomic presence of *APF2*::*GFP*, encoding a GFP-tagged version of the apicidin F transcription factor. For overexpressing *APF2*::*GFP* via constitutive *OLIC* promoter from *A. nidulans*, *ΔAPF2*/OE::*APF2*::*GFP* transformants (T) with ectopic integration were identified via diagnostic PCR using primer pair PoliC-seqF2/OgfpC-seqR1 (1.99 kb). DNA were utilised as positive and negative control, respectively; M =  GeneRuler 1 kb Plus DNA Ladder, V =  vector: pOE::*APF2*::*GFP* and WT =  wild type. Figure S2. The transcription factor (TF) Apf2 is localized in the nucleus. It was fused to green fluorescent protein (GFP) at the C-terminus. The Δ*APF2* mutant was used as background. Δ*APF2*/OE::*APF2*::*GFP* and Δ*APF2* as a control were applied for epifluorescene microscopy. The two strains were grown for one day in 60 mM glutamine. The nuclei were stained with the fluorescent dye Hoechst 33342 and analyzed with the DAPI filter set. Size of scale bars is indicated. Figure S3. Mutation of putative Apf2 binding site upstream of *APF1*. (A) The strategy of the point mutations in the promoter region of *APF1* is depicted here. While the wild type (WT) motif was followed by an interrupted key gene, full length *APF1* was preceded by two versions of mutated motifs, designated “P-mut1” and “P-mut2”; *NAT1* =  nourseothricin resistance cassette, AMP =  ampicillin resistance, URA =  uracil prototrophy. (B) It was screened for transformants with *in locus* integration of the vectors that contained mutated *APF1* promoter sequences and additionally, 1.5 kb of *APF1*. *In locus* integration of pProm::*APF1*::P-mut1 (T1, T2) and pProm::*APF1*::P-mut2 (T3) was analysed with primer combination pCSN44-trpCP3/00003_apf1_OE_R (2.69 kb) while WT signal was obtained using 00004_apf11_5R/00003_apf1_OE_R (2.67 kb). Additionally, WT DNA was utilised as a negative control; M =  GeneRuler 1 kb Plus DNA Ladder; T =  transformant. Figure S4. Deletion strategy and Southern blot of the wild-type (WT) and two independent *APF1* deletion mutants (NRPS). (A) The Δ*APF1* mutants have the hygromycin resistance gene (*HPH*). The 5′ and the 3′ flanks are depicted with the shaded area. Genomic DNA of the two mutants and the WT was digested with BamHI. The 5′ flank was used as probe in the Southern blot. The WT-allele (∼3.3 kb) is absent in T3 and T4. The Gene Ruler DNA ladder mix was used as marker. Figure S5. Deletion strategy and Southern blot of the wild type (WT) and three independent *APF2* deletion mutants (transcription factor). (A) The Δ*APF2* mutants have the hygromycin resistance gene (*HPH*). The 5′ and the 3′ flanks are depicted with the shaded area. Genomic DNA of the three mutants and the WT was digested with ScaI. The 3′ flank was used as probe in the Southern blot. The WT-allele (∼10.0 kb) is absent in T3, T5 and T9. The Gene Ruler DNA ladder mix was used as marker. Figure S6. Deletion strategy and Southern blot of the wild type (WT) and three independent *APF3* deletion mutants (Δ^1^-pyrroline-5-carboxylate reductase). (A) The Δ*APF3* mutants have the hygromycin resistance gene (*HPH*). The 5′ and the 3′ flanks are depicted with the shaded area. Genomic DNA of the three mutants and the WT was digested with NdeI. The 5′ flank was used as probe in the Southern blot. The WT-allele (∼3.3 kb) is absent in T1, T3 and T4. The Gene Ruler DNA ladder mix was used as marker. Figure S7. Deletion strategy and Southern blot of the wild type (WT) and three independent *APF6* deletion mutants (*O*-methyltransferase). (A) The Δ*APF6* mutants have the hygromycin resistance gene (*HPH*). The 5′ and the 3′ flanks are depicted with the shaded area. Genomic DNA of the three mutants and the WT was digested with EcoRI. The 3′ flank was used as probe in the Southern blot. The WT-allele (∼3.0 kb) is absent in T1, T2 and T4. The Gene Ruler DNA ladder mix was used as marker. Mutant T2 has an additional ectopic integration. For analyses the other mutants were used. Figure S8. Deletion strategy and Southern blot of the wild type (WT) and three independent *APF9* deletion mutants (FAD-dependent monooxygenase). (A) The Δ*APF9* mutants have the hygromycin resistance gene (*HPH*). The 5′ and the 3′ flanks are depicted with the shaded area. Genomic DNA of the three mutants and the WT was digested with ScaI. The 5′ flank was used as probe in the Southern blot. The WT-allele (∼10.7 kb) is absent in T2, T4 and T8. The Gene Ruler DNA ladder mix was used as marker. Figure S9. Deletion strategy and Southern blot of the wild type (WT) and two independent *APF11* deletion mutants (major facilitator superfamily transporter). (A) The Δ*APF11* mutants have the nourseothricin resistance gene (*NAT*). The 5′ and the 3′ flanks are depicted with the shaded area. Genomic DNA of the two mutants and the WT was digested with SpeI. The 5′ flank was used as probe in the southern blot. The WT-allele (∼4.0 kb) is absent in T4 and T5. The Gene Ruler DNA ladder mix was used as marker. Figure S10. Comparative HPLC-HRMS-analysis of the mycelium extracts of the wild type (WT) and the single deletion mutants of the *APF* gene cluster. The different strains were grown in ICI with 60 mM glutamine for three days. Shown are the extracted ion chromatograms for the [M+H]^+^-ion of apicidin F (APF) (646.3235±0.0032), the axes are normalized to the wild-type level. In the mutants Δ*APF3*, Δ*APF6* and Δ*APF11* apicidin F was still detected. Figure S11. HPLC-HRMS of the marfey's derivatives of apicidin J hydrolysate and standard amino acids. HESI positive mode *m/z* 100-700, shown are the extracted ion chromatograms of the different amino acid derivatives normalized to the largest peak. Figure S12. Partial hydrolysis and sequences of the di- and tripeptides. (A) Tri- and dipeptidic compounds resulting from partial hydrolysis of apicidin F analyzed by HPLC-HRMS. Shown is the TIC from *m/z* 50 to 700. (B) Possible sequences of apicidin J compared to di-and tripeptides produced by hydrolysis. (C) Two possible structures of apicidin J. (D) MS^2^ fragmentation (CID 35.0%) of the tripeptide with *m/z* 489.23 compared to the backbone fragmentation of possible tripeptide structures. (E) (CID 35.0%) of the tripeptide with *m/z* 434.23 compared to the backbone fragmentation of possible tripeptide structures. Data evaluation has been done as described in von Bargen et al., 2013. Figure S13. ^1^H-NMR (400 MHz, C_5_D_5_N) spectrum of apicidin K. Figure S14. ^13^C-NMR (400 MHz, C_5_D_5_N) spectrum of apicidin K. Figure S15. H, H-COSY-NMR (400 MHz, C_5_D_5_N) spectrum of apicidin K. Figure S16. HSQC (400 MHz, C_5_D_5_N) spectrum of apicidin K. Figure S17. HMBC (400 MHz, C_5_D_5_N) spectrum of apicidin K. Figure S18. HPLC-HRMSof the marfey's derivatives of apicidin K hydrolysate and standard amino acids. HESI positive mode *m/z* 100–700, shown are the extracted ion chromatograms of the different amino acid derivatives normalized to the largest peak. Table S1. List of all primers used in this study. Table S2. NMR Spectroscopic Data (400 MHz, C_5_D_5_N) for apicidin K.
(DOCX)
