# Supplementary material for: Apicidin F: Characterization and Genetic Manipulation of a New Secondary Metabolite Gene Cluster in the Rice Pathogen Fusarium fujikuroi
Source: PLoS One. 2014 Jul 24;9(7):e103336. doi: 10.1371/journal.pone.0103336 (PMC4109984; doi:10.1371/journal.pone.0103336)
Supplement: File S1 — Supporting figures and tables. Figure S1. Verification of genomic presence of APF2::GFP, encoding a GFP-tagged version of the apicidin F transcription factor. For overexpressing APF2::GFP via constitutive OLIC promoter from A. nidulans, ΔAPF2/OE::APF2::GFP transformants (T) with ectopic integration were identified via diagnostic PCR using primer pair PoliC-seqF2/OgfpC-seqR1 (1.99 kb). DNA were utilised as positive and negative control, respectively; M = GeneRuler 1 kb Plus DNA Ladder, V = vector: pOE::APF2::GFP and WT = wild type. Figure S2. The transcription factor (TF) Apf2 is localized in the nucleus. It was fused to green fluorescent protein (GFP) at the C-terminus. The ΔAPF2 mutant was used as background. ΔAPF2/OE::APF2::GFP and ΔAPF2 as a control were applied for epifluorescene microscopy. The two strains were grown for one day in 60 mM glutamine. The nuclei were stained with the fluorescent dye Hoechst 33342 and analyzed with the DAPI filter set. Size of scale bars is indicated. Figure S3. Mutation of putative Apf2 binding site upstream of APF1. (A) The strategy of the point mutations in the promoter region of APF1 is depicted here. While the wild type (WT) motif was followed by an interrupted key gene, full length APF1 was preceded by two versions of mutated motifs, designated “P-mut1” and “P-mut2”; NAT1 = nourseothricin resistance cassette, AMP = ampicillin resistance, URA = uracil prototrophy. (B) It was screened for transformants with in locus integration of the vectors that contained mutated APF1 promoter sequences and additionally, 1.5 kb of APF1. In locus integration of pProm::APF1::P-mut1 (T1, T2) and pProm::APF1::P-mut2 (T3) was analysed with primer combination pCSN44-trpCP3/00003_apf1_OE_R (2.69 kb) while WT signal was obtained using 00004_apf11_5R/00003_apf1_OE_R (2.67 kb). Additionally, WT DNA was utilised as a negative control; M = GeneRuler 1 kb Plus DNA Ladder; T = transformant. Figure S4. Deletion strategy and Southern blot of the wild-type (W [file pone.0103336.s001.docx]

**Supporting information**

**Apicidin F: Characterization and genetic manipulation of a new secondary metabolite gene cluster in the rice pathogen *Fusarium fujikuroi***

Eva-Maria Niehaus^1,†^, Slavica Janevska^1,†^, Katharina W. von Bargen^2,†^, Christian M. K. Sieber^3^, Henning Harrer^2^, Hans-Ulrich Humpf^2,^*, Bettina Tudzynski^1,^*

^1^Institut für Biologie und Biotechnologie der Pflanzen, Westfälische Wilhelms-Universität Münster, Schlossplatz 8, D-48143 Münster

^2^Institut für Lebensmittelchemie, Westfälische Wilhelms-Universität Münster, Corrensstr. 45, D-48149 Münster

^3^Institut für Bioinformatik und Systembiologie, Helmholtz Zentrum München (GmbH), Ingolstädter Landstr. 1, D-85764 Neuherberg

*Prof. Dr. B. Tudzynski, fon: +49 251 83 24801, fax: +49 251 83 21601, e-mail: tudzynsb@uni-muenster.de

*Prof. Dr. H.-U. Humpf, fon: +49 251 83 33391, fax: +49 251 83 33396, e-mail: humpf@uni-muenster.de

† authors contributed equally

**Supplementary Figures**


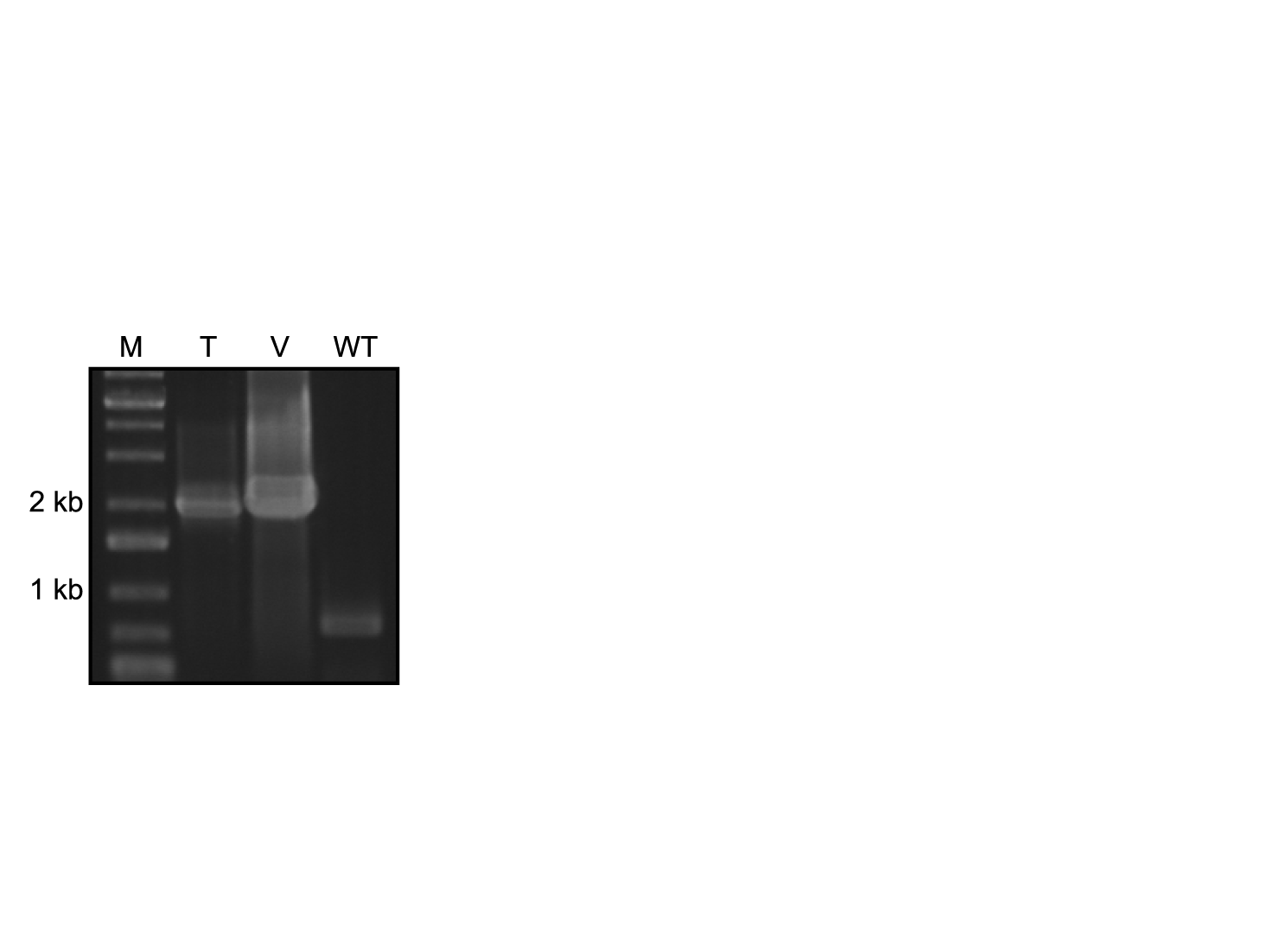


Fig. S1: Verification of genomic presence of *APF2*::*GFP*, encoding a GFP-tagged version of the apicidin F transcription factor. For overexpressing *APF2*::*GFP* via constitutive *OLIC* promoter from *A. nidulans*, *ΔAPF2*/OE::*APF2*::*GFP* transformants (T) with ectopic integration were identified via diagnostic PCR using primer pair PoliC-seqF2/OgfpC-seqR1 (1.99 kb). DNA were utilised as positive and negative control, respectively; M = GeneRuler 1 kb Plus DNA Ladder, V = vector: pOE::*APF2*::*GFP* and WT = wild type.


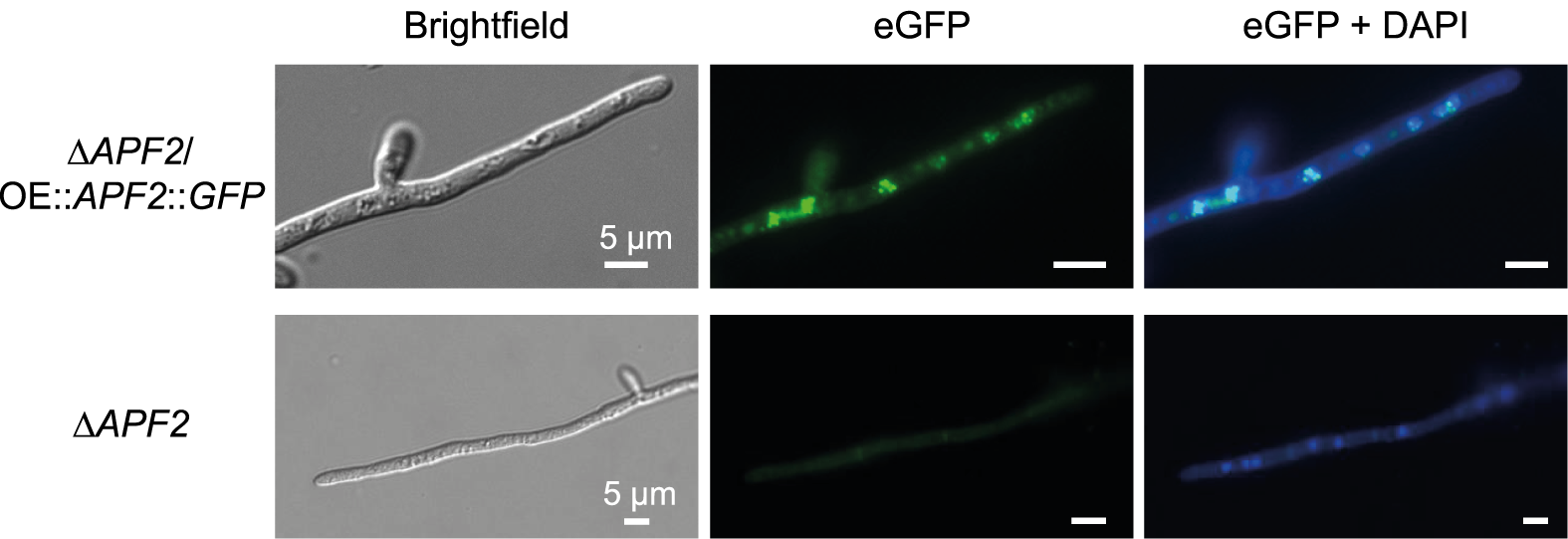


**Fig. S2:** **The transcription factor (TF) Apf2 is localized in the nucleus.** It was fused to green fluorescent protein (GFP) at the C-terminus. The ∆*APF2* mutant was used as background. ∆*APF2*/OE::*APF2*::*GFP* and ∆*APF2* as a control were applied for epifluorescene microscopy. The two strains were grown for one day in 60 mM glutamine. The nuclei were stained with the fluorescent dye Hoechst 33342 and analyzed with the DAPI filter set. Size of scale bars is indicated.


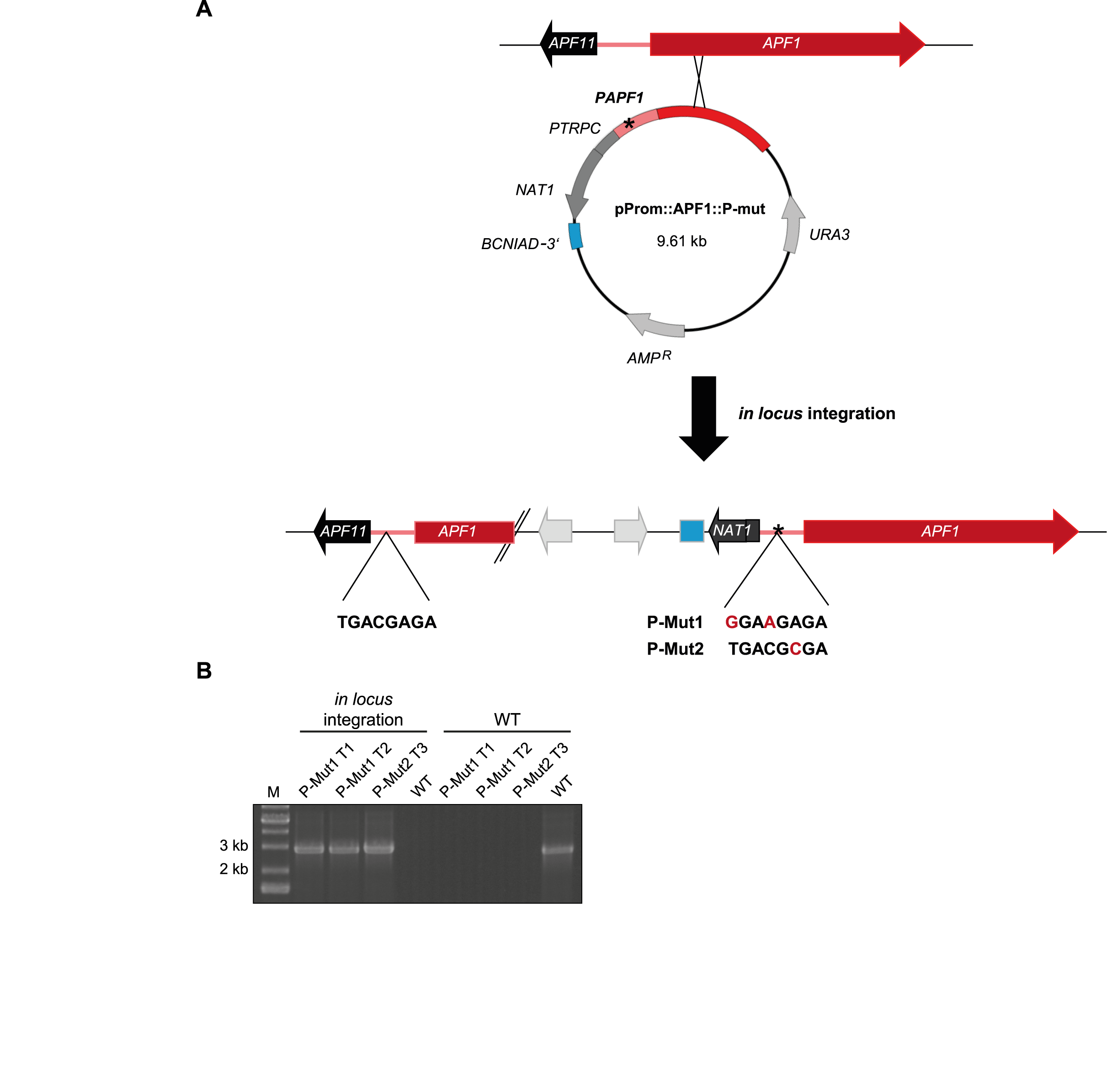


Fig. S3: Mutation of putative Apf2 binding site upstream of *APF1*. (A) The strategy of the point mutations in the promoter region of *APF1* is depicted here. While the wild type (WT) motif was followed by an interrupted key gene, full length *APF1* was preceded by two versions of mutated motifs, designated “P-mut1” and “P-mut2”; *NAT1* = nourseothricin resistance cassette, AMP = ampicillin resistance, URA = uracil prototrophy. (B) It was screened for transformants with *in locus* integration of the vectors that contained mutated *APF1* promoter sequences and additionally, 1.5 kb of *APF1*. *In locus* integration of pProm::*APF1*::P-mut1 (T1, T2) and pProm::*APF1*::P-mut2 (T3) was analysed with primer combination pCSN44-trpCP3/00003_apf1_OE_R (2.69 kb) while WT signal was obtained using 00004_apf11_5R/00003_apf1_OE_R (2.67 kb). Additionally, WT DNA was utilised as a negative control; M = GeneRuler 1 kb Plus DNA Ladder; T = transformant.


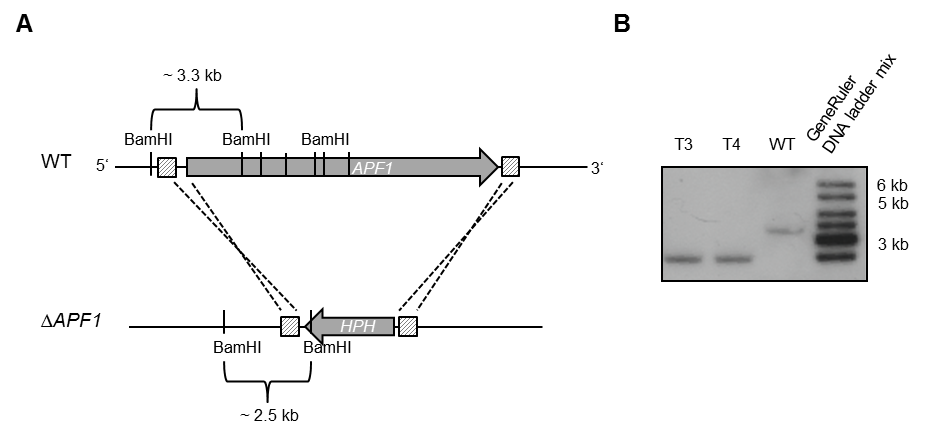


**Fig. S4: Deletion strategy and Southern blot of the wild-type (WT) and two independent *APF1* deletion mutants (NRPS).** (A) The ∆*APF1* mutants have the hygromycin resistance gene (*HPH*). The 5’ and the 3’ flanks are depicted with the shaded area. Genomic DNA of the two mutants and the WT was digested with BamHI. The 5’ flank was used as probe in the Southern blot. The WT-allele (~ 3.3 kb) is absent in T3 and T4. The Gene Ruler DNA ladder mix was used as marker.


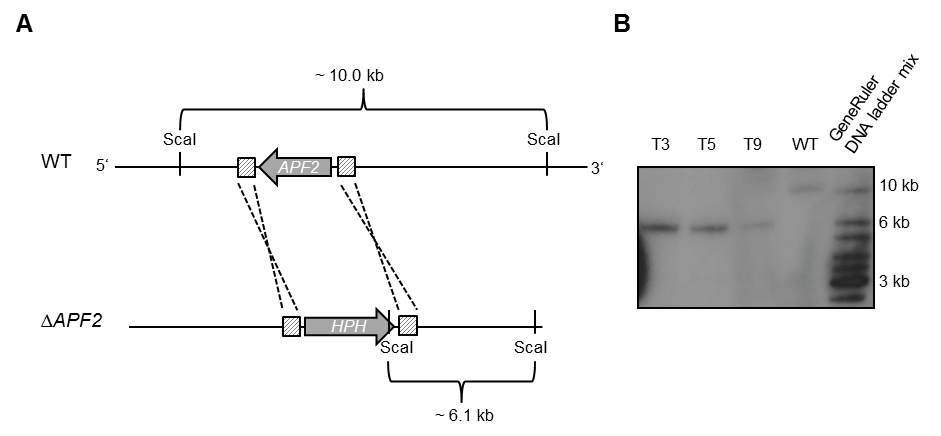


**Fig. S5: Deletion strategy and Southern blot of the wild type (WT) and three independent *APF2* deletion mutants (transcription factor).** (A) The ∆*APF2* mutants have the hygromycin resistance gene (*HPH*). The 5’ and the 3’ flanks are depicted with the shaded area. Genomic DNA of the three mutants and the WT was digested with ScaI. The 3’ flank was used as probe in the Southern blot. The WT-allele (~ 10.0 kb) is absent in T3, T5 and T9. The Gene Ruler DNA ladder mix was used as marker.


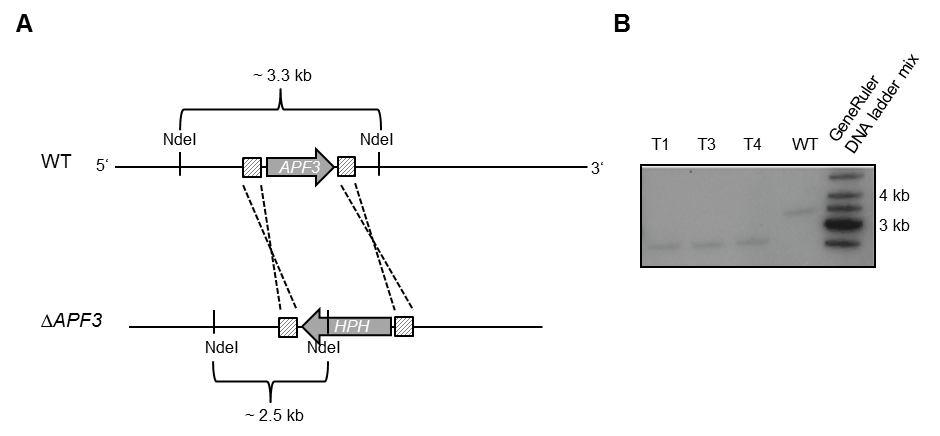


**Fig. S6: Deletion strategy and Southern blot of the wild type (WT) and three independent *APF3* deletion mutants (∆^1^-pyrroline-5-carboxylate reductase).** (A) The ∆*APF3* mutants have the hygromycin resistance gene (*HPH*). The 5’ and the 3’ flanks are depicted with the shaded area. Genomic DNA of the three mutants and the WT was digested with NdeI. The 5’ flank was used as probe in the Southern blot. The WT-allele (~ 3.3 kb) is absent in T1, T3 and T4. The Gene Ruler DNA ladder mix was used as marker.


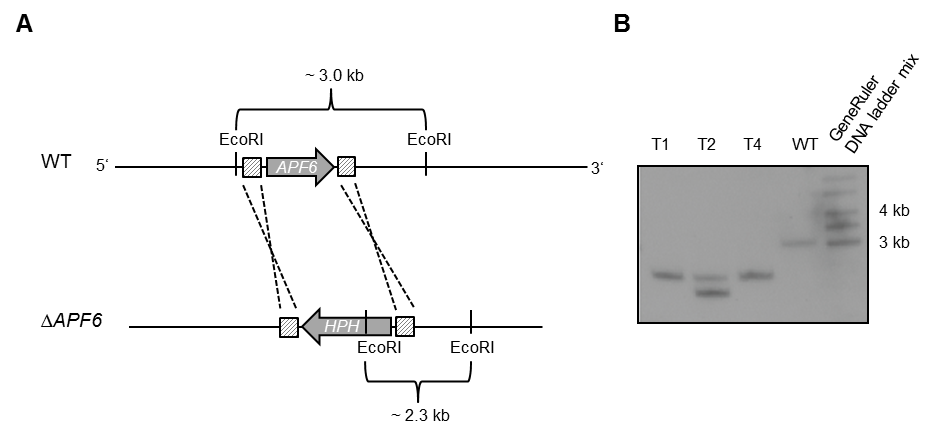


**Fig. S7:** **Deletion strategy and Southern blot of the wild type (WT) and three independent *APF6* deletion mutants (*O*-methyltransferase).** (A) The ∆*APF6* mutants have the hygromycin resistance gene (*HPH*). The 5’ and the 3’ flanks are depicted with the shaded area. Genomic DNA of the three mutants and the WT was digested with EcoRI. The 3’ flank was used as probe in the Southern blot. The WT-allele (~ 3.0 kb) is absent in T1, T2 and T4. The Gene Ruler DNA ladder mix was used as marker. Mutant T2 has an additional ectopic integration. For analyses the other mutants were used.


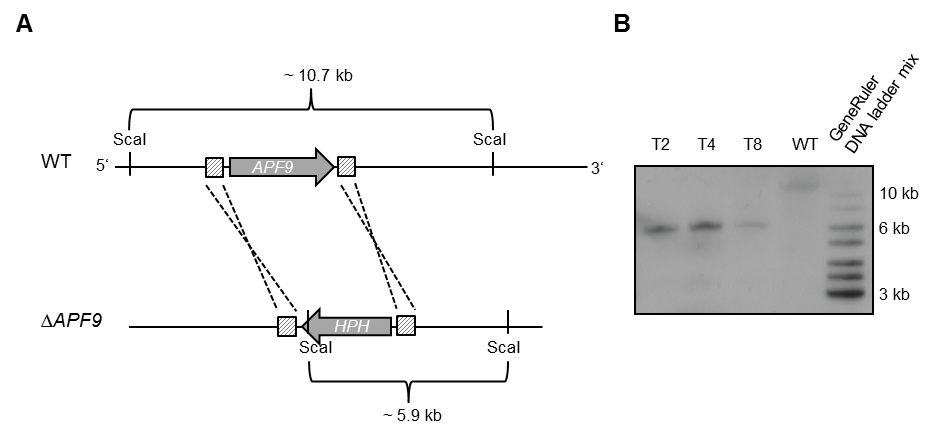


**Fig. S8: Deletion strategy and Southern blot of the wild type (WT) and three independent *APF9* deletion mutants** (**FAD-dependent monooxygenase).** (A) The ∆*APF9* mutants have the hygromycin resistance gene (*HPH*). The 5’ and the 3’ flanks are depicted with the shaded area. Genomic DNA of the three mutants and the WT was digested with ScaI. The 5’ flank was used as probe in the Southern blot. The WT-allele (~ 10.7 kb) is absent in T2, T4 and T8. The Gene Ruler DNA ladder mix was used as marker.


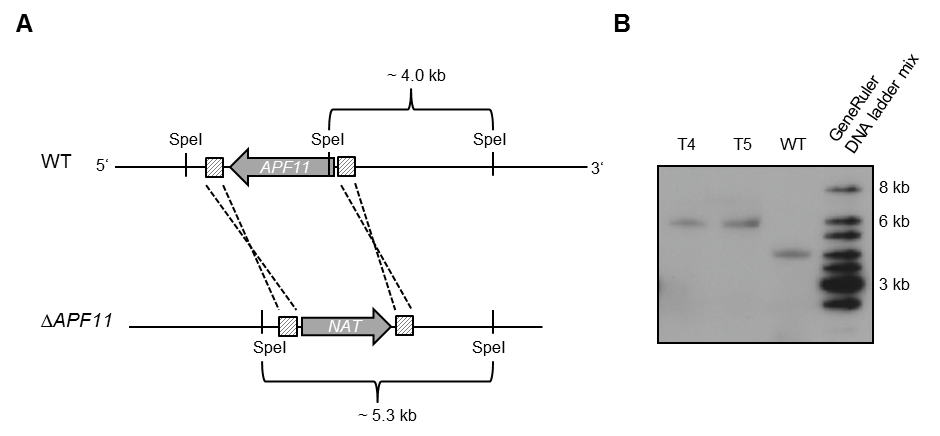


**Fig. S9: Deletion strategy and Southern blot of the wild type (WT) and two independent *APF11* deletion mutants (major facilitator superfamily transporter).** (A) The ∆*APF11* mutants have the nourseothricin resistance gene (*NAT*). The 5’ and the 3’ flanks are depicted with the shaded area. Genomic DNA of the two mutants and the WT was digested with SpeI. The 5’ flank was used as probe in the southern blot. The WT-allele (~ 4.0 kb) is absent in T4 and T5. The Gene Ruler DNA ladder mix was used as marker.


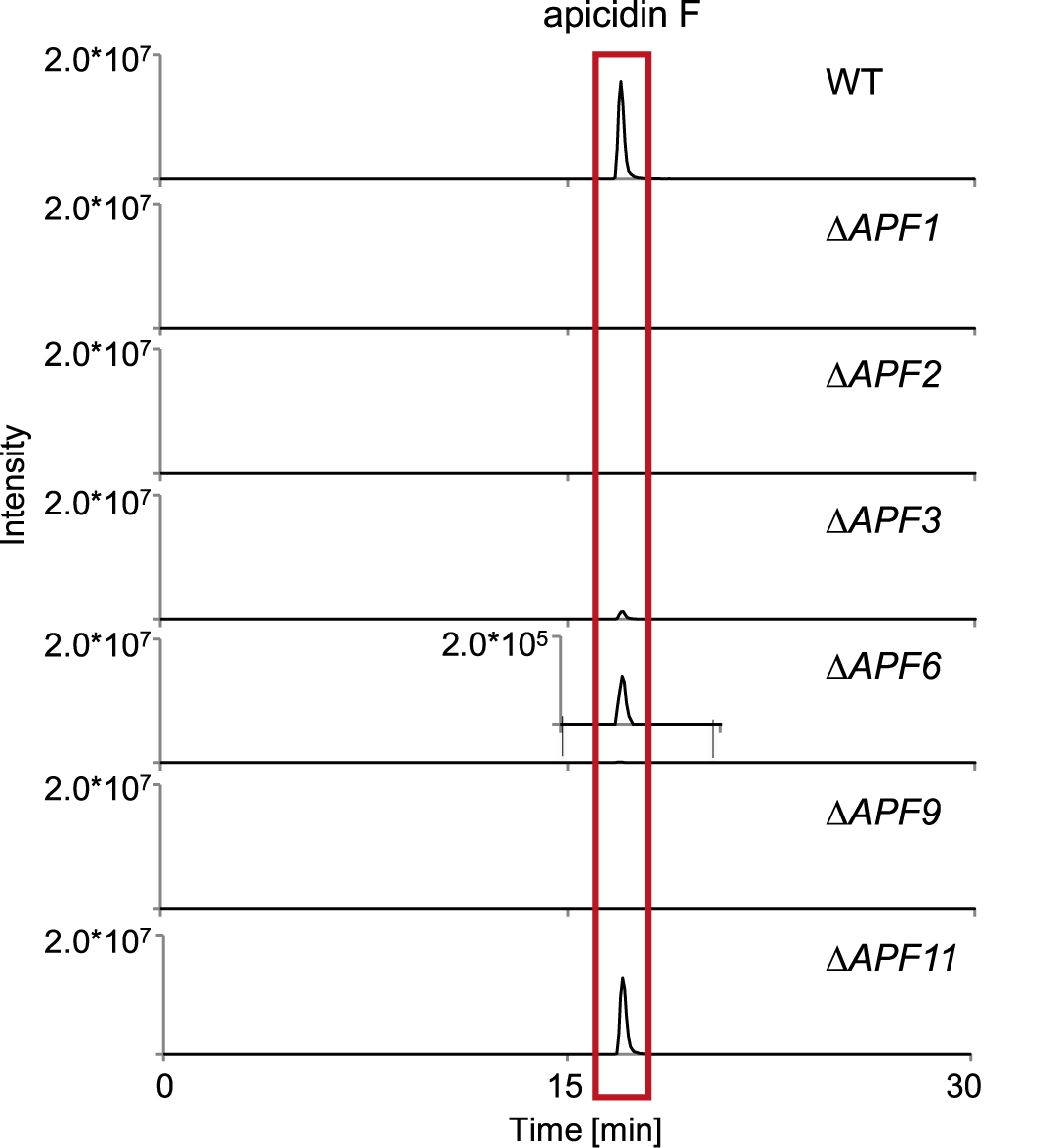


Fig. S10: Comparative HPLC-HRMS-analysis of the mycelium extracts of the wild type (WT) and the single deletion mutants of the *APF* gene cluster. The different strains were grown in ICI with 60 mM glutamine for three days. Shown are the extracted ion chromatograms for the [M+H]^+^-ion of apicidin F (APF) (646.3235 ± 0.0032), the axes are normalized to the wild-type level. In the mutants ∆*APF3*, ∆*APF6* and ∆*APF11* apicidin F was still detected.


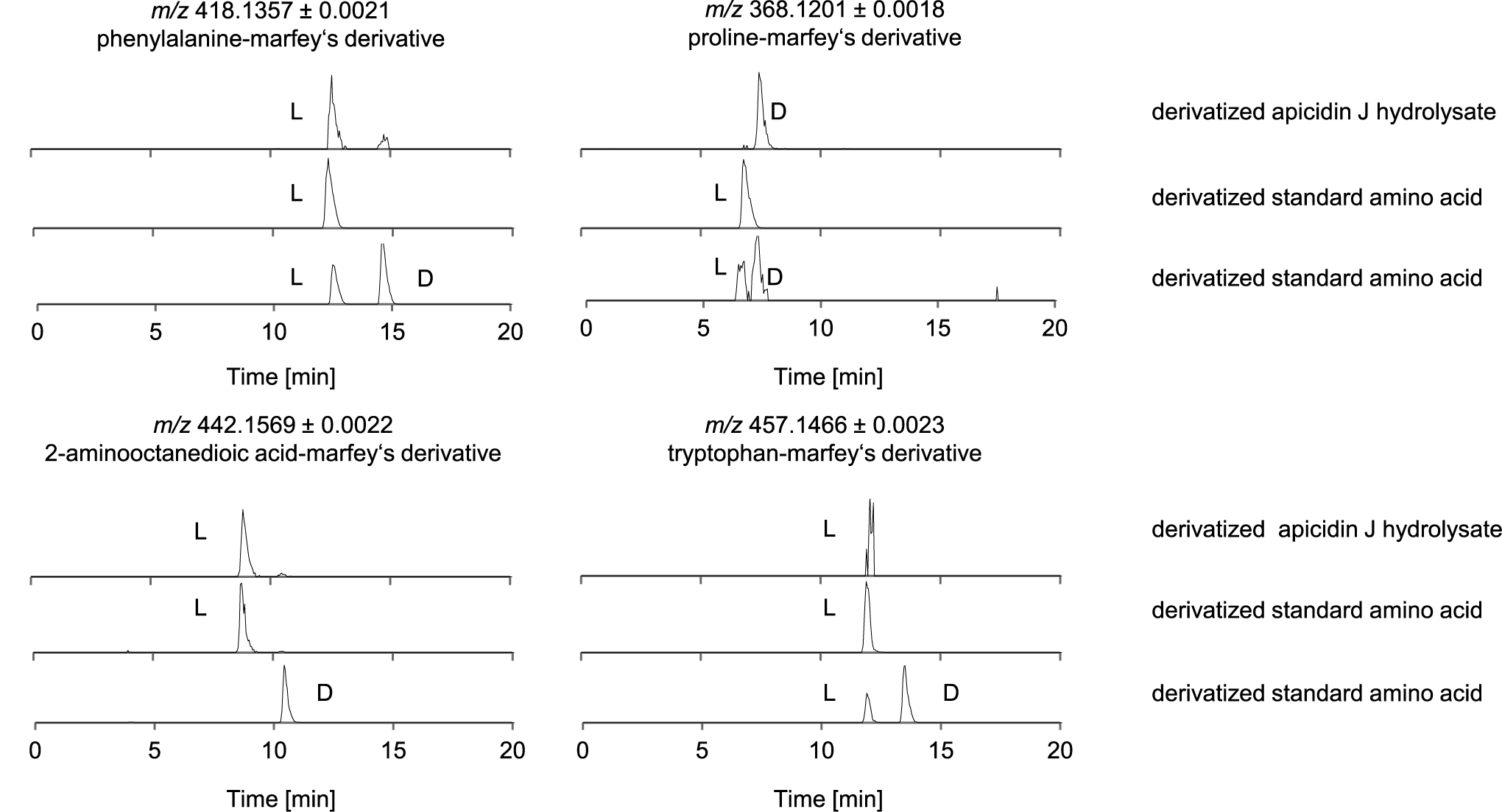


Fig. S11: HPLC-HRMS of the marfey’s derivatives of apicidin J hydrolysate and standard amino acids. HESI positive mode *m/z* 100-700, shown are the extracted ion chromatograms of the different amino acid derivatives normalized to the largest peak.

Fig. S12: Partial hydrolysis and sequences of the di- and tripeptides. (A) Tri- and dipeptidic compounds resulting from partial hydrolysis of apicidin F analyzed by HPLC-HRMS. Shown is the TIC from *m/z* 50 to 700. (B) Possible sequences of apicidin J compared to di-and tripeptides produced by hydrolysis. (C) Two possible structures of apicidin J. (D) MS^2^ fragmentation (CID 35.0%) of the tripeptide with *m/z* 489.23 compared to the backbone fragmentation of possible tripeptide structures. (E) (CID 35.0%) of the tripeptide with *m/z* 434.23 compared to the backbone fragmentation of possible tripeptide structures. Data evaluation has been done as described in von Bargen et al., 2013.

Fig. S13: ^1^H-NMR (400 MHz, C_5_D_5_N) spectrum of apicidin K.

**Fig. S14: ^13^C-NMR (400 MHz, C_5_D_5_N) spectrum of apicidin K.**

**Fig. S15: H, H-COSY-NMR (400 MHz, C_5_D_5_N) spectrum of apicidin K.**

**Fig. S16: HSQC (400 MHz, C_5_D_5_N) spectrum of apicidin K.**

**Fig. S17: HMBC (400 MHz, C_5_D_5_N) spectrum of apicidin K.**


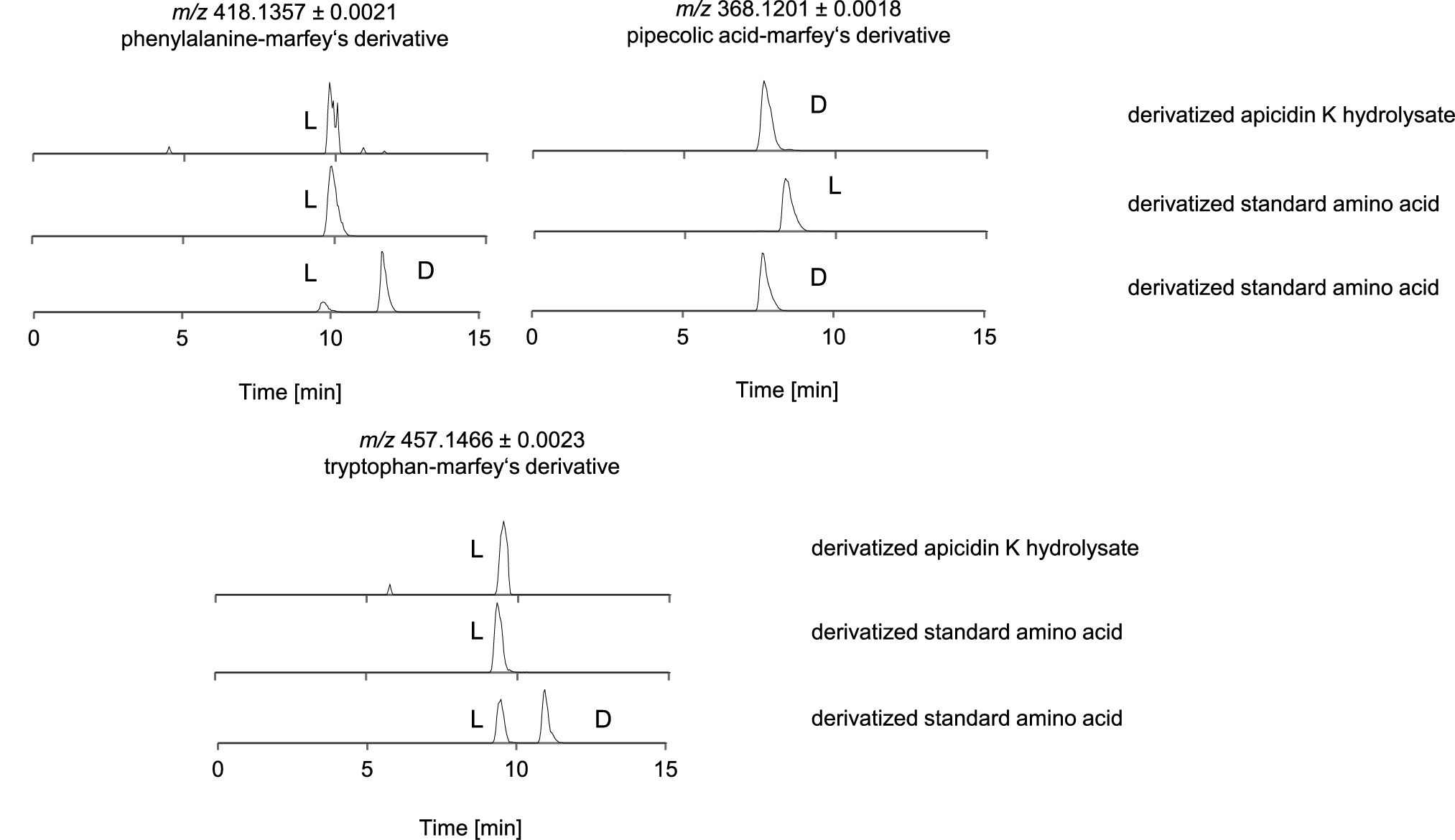


Fig. S18: HPLC-HRMS of the marfey’s derivatives of apicidin K hydrolysate and standard amino acids. HESI positive mode *m/z* 100-700, shown are the extracted ion chromatograms of the different amino acid derivatives normalized to the largest peak.

**Tables**

**Table S1: List of all primers used in this study.**

| **Primer** | **Sequence** | |
| --- | --- | --- |
| **Amplification of the gene flanks** | | |
| apf1-5F | GTAACGCCAGGGTTTTCCCAGTCACGACGGCCGAGCCATACCAGGCG | |
| apf1-5R | ATCCACTTAACGTTACTGAAATCTCCAACCGCTTGCAGTGTGAGTGAATGC | |
| apf1-3F | CTCCTTCAATATCATCTTCTGTCTCCGACGGAGAAGCCGTCGTTGCCAAC | |
| apf1-3R | GCGGATAACAATTTCACACAGGAAACAGCTGGCCTAGATATTGCAGCCTGG | |
| apf2-5F | GTAACGCCAGGGTTTTCCCAGTCACGACGCCGCTCGAGTTCGGTTCCAG | |
| apf2-5R | ATCCACTTAACGTTACTGAAATCTCCAACGTGCAGCCATGGCACACGC | |
| apf2-3F | CTCCTTCAATATCATCTTCTGTCTCCGACCAGCGGGCCTGTGAAGTACG | |
| apf2-3R | GCGGATAACAATTTCACACAGGAAACAGCCACGGCACGCAGGTCTGC | |
| apf3-5F | GTAACGCCAGGGTTTTCCCAGTCACGACGCGCATCAACAGACCTGCACTCAC | |
| apf3-5R | ATCCACTTAACGTTACTGAAATCTCCAACGTCCAAATTGGATGATGTTGATGG | |
| apf3-3F | CTCCTTCAATATCATCTTCTGTCTCCGACCCGAGATGAAGAAGGACAGGCC | |
| apf3-3R | GCGGATAACAATTTCACACAGGAAACAGCGAAGCGCTGGCTTGAGACACC | |
| apf6-5F | GTAACGCCAGGGTTTTCCCAGTCACGACGGCACCAACTGGCTCCATTGAGC | |
| apf6-5R | ATCCACTTAACGTTACTGAAATCTCCAACTGAGACCGTTGATCGACGTTGG | |
| apf6-3F | CTCCTTCAATATCATCTTCTGTCTCCGACGCCGCCTGCATTATTGTAGCC | |
| apf6-3R | GCGGATAACAATTTCACACAGGAAACAGCATCAGGATGCCAAGGTCGACG | |
| apf9-5F | GTAACGCCAGGGTTTTCCCAGTCACGACGGGAGATCGGACCAGGCGG | |
| apf9-5R | ATCCACTTAACGTTACTGAAATCTCCAACTTGGCAGTGCAAGCCGCC | |
| apf9-3F | CTCCTTCAATATCATCTTCTGTCTCCGACCTCATCGGTCTTCAAGAGCGCG | |
| apf9-3R | GCGGATAACAATTTCACACAGGAAACAGCGTTGATTCGCTGGATCCCGC | |
| apf11-5F | GTAACGCCAGGGTTTTCCCAGTCACGACGCGCTTGCAGTGTGAGTGAATGC | |
| apf11-5R | ATCCACTTAACGTTACTGAAATCTCCAACCGTGGCGGCGCTGATATCC | |
| apf11-3F | CTCCTTCAATATCATCTTCTGTCTCCGACCTGAGAAGGGCACGGTTGTCC | |
| apf11-3R | GCGGATAACAATTTCACACAGGAAACAGCGGTCCCACTGGGACAGATTGC | |
| **Diagnostic primers** | | |
| apf1-5F-diag | GGTGAGCAGGAGCCAGGAGC | |
| apf1-3R-diag | GCCAGGTTAACTTCTTTAAGGTTGC | |
| apf1-WT-F | GGAGATACAATTGCCGG | |
| apf1-WT-R | GCGTCGATGGCGGTGTGTGACG | |
| apf2-5F-diag | GCTTGCGTTCACATGGCCG | |
| apf2-3R-diag | GCGTTATGGCACGCTGCATG | |
| apf2-WT-F | ACAGACGCCAATGAACGCCG | |
| apf2-WT-R | CCACGGCTCTGTGCCGC | |
| apf3-5F-diag | GATGGCGAGGCATGGTGTTG | |
| apf3-3R-diag | ACAGACGCCAATGAACGCCG | |
| apf3-WT-F | CACCAGCGATGTGTGCCAGG | |
| apf3-WT-R | CTTGCATAGCAGCTCCGAGGC | |
| apf6-5F-diag | CCGTCTGGACTCGGCGAGG | |
| apf6-3R-diag | GACGCGCGTCGCAAGGC | |
| apf6-WT-F | CTCCAGATCATGAGCGCCTCC | |
| apf6-WT-R | CTCGCAATCCGCATCTGGC | |
| apf9-5F-diag | GGACCTGAGCTGAACTCTTGCG | |
| apf9-3R-diag | CGATCGGCACACTCTCCTTAGC | |
| apf9-WT-F | CCTCATTGGAGGAGTGGTCACG | |
| apf9-WT-R | GGACATGTCGCCAGCACAGG | |
| apf11-5F-diag | CGCAGTGTCACGCACCAAGC | |
| apf11-3R-diag | CCACCGATGTTGACGCCTACG | |
| apf11-WT-F | CCGTCTGGACTCGGCGAGG | |
| apf11-WT-R | CCCTCCACTAGCCGTTCCACG | |
| **Diagnostic primers for the resistance cassette** | | |
| pCSN44-hph-trpC-T | | GGAATAGAGTAGATGCCGACCGG |
| pCSN44-trpC-P2 | | GTGATCCGCCTGGACGACTAAACC |
| nat1-seqF1 | | CGGACGGCGAGCGGCAGGCGC |
| **Wild type primers of the border gene of the apicidin F cluster** | | |
| FFUJ_00014-F | GCCAGCTGAATGGCGTCAGG | |
| FFUJ_00014-R | CACGCTGCTACGGCCGGC | |
| **Amplification of the resistance cassette** | | |
| hphF | GTCGGAGACAGAAGATGATATTGAAGGAGC | |
| hphR | GTTGGAGATTTCAGTAACGTTAAGTGGAT | |
| **Wild type primers for the other apicidin F cluster genes** | | |
| apf4_WT_F | GCTTGCGTTCACATGGCCG | |
| apf4_WT_R | GCACGGTCTGATGGTTGCTGC | |
| apf5_WT_F | CCGAGCTATTCCCATGACTGGC | |
| apf5_WT_R | CGCGGCTGATTGCACAGATCC | |
| apf7_WT_F | GACGCGCGTCGCAAGGC | |
| apf7_WT_R | CCGACCTCACAGCCACCAGG | |
| apf8_WT_F | GCCTGATAGACATGGCG | |
| apf8_WT_R | CGCGACTATAGCATTTGC | |
| apf12_WT_F | CGCAGTGTCACGCACCAAGC | |
| apf12_WT_R | CCCTCCACTAGCCGTTCCACG | |
| **Gfp-primers for *APF2*** | | |
| apf2_gfp_OE_F | CCATCACATCACAATCGATCCAACCATGTCGCCACCAAGT | |
| apf2_gfp_OE_R | TACTTACCTCACCCTTGGAAACCATGTCACAACCAATATT | |
| apf2_gfp_seq | ATGTCAGCAGTGGTGGTGAGAG | |
| OliC-P-seqF1 | CCTTTCCCATCATCCATCTCCTC | |
| Ogfp-seqR1 | CGTCTCCCTCACCCTCTCCG | |
| PoliC-seqF2 | GGGAGACGTATTTAGGTGCTAGGG | |
| OgfpC-seqR1 | CTGCCAATTGAACAGAGCCATCC | |
| **Primers for the mutation of the promoter of *APF1*** | | |
| Prom_apf1_F | GCCCAAAAAATGCTCCTTCAATATCGCTGCAGGTATCTCAGCAG | |
| Papf1_mut1_R | CTCAGCTCTCTTCCGCGCGCGAGGC | |
| Papf1_mut2_R | CTCAGCTCGCGTCAGCGCGCGAGGC | |
| Prom_apf1_R | GTAACGCCAGGGTTTTCCCAGTCACGACGCTGCTTGCAGTCCGTTCAGG | |
| Papf1_mut1_F | GCCTCGCGCGCGGAAGAGAGCTGAG | |
| Papf1_mut2_F | GCCTCGCGCGCTGACGCGAGCTGAG | |
| Prom_apf1_seq | CCAATTTGAATTTGGGCTTGCC | |
| pCSN44-trpC-P3 | CTAATAAGAGTCACACTTCGAGC | |
| trpC-P-seqR1 | CATTGTTGACCTCCACTAGCTCC | |

Table S2: NMR Spectroscopic Data (400 MHz, C_5_D_5_N) for apicidin K

|  |  | **C_5_D_5_N** | |
| --- | --- | --- | --- |
| **position** |  | **δ_C_, type** | **δ_H_ (J in Hz)** |
| ***N*-methoxytyprophan** | | | |
| NH |  |  | 10.03, d (6.8) |
| 1 |  | 174.2^1^, C | - |
| 2 |  | 61.9, CH | 4.54, dt (9.9, 6.8) |
| 3 |  | 26.6^2^, CH_2_ | 4.22-4.08, m  3.76-3.73, m |
| 4 |  | 108.5, C | - |
| 4a |  | 124.5, C | - |
| 5 |  | 119.8, CH | 7.73, d (7.9) |
| 6 |  | 120.6, CH | 7.20-7.15, m |
| 7 |  | 123.2, CH | 7.29-7.25, m |
| 8 |  | 109.2, CH | 7.54, d (8.2) |
| 8a |  | 133.3, C | - |
| 10 |  | 123.3, CH | 7.43, s |
| OCH_3_ |  | 66.1, CH_3_ | 3.92, s |
| **2-amino-8-hydroxy-octanoic acid** | | | |
| NH |  | - | 7.37-7.31, m |
| 1 |  | 177.1, C | - |
| 2 |  | 55.4, CH | 4.79, q (8.5) |
| 3 |  | 30.7, CH_2_ | 1.98-1.90, m  1.73-1.54, m |
| 4 |  | 26.5^2^, CH_2_ | 1.31-1.16, m |
| 5 |  | 29.7, CH_2_ | 1.31-1.16, m |
| 6 |  | 26.5^2^, CH_2_ | 1.47-1.31, m |
| 7 |  | 33.9, CH_2_ | 1.73-1.54, m |
| 8 |  | 62.3, CH_2_ | 3.80, t (6.6) |
| **pipecolic acid** | | | |
| 1 |  | 172.4, C | - |
| 2 |  | 51.4, CH | 5.47, d (5.2) |
| 3 |  | 24.9, CH_2_ | 2.02-2.00, m  1.31-1.16, m |
| 4 |  | 20.3, CH_2_ | 2.39-2.21, m  1.47-1.31, m |
| 5 |  | 25.9, CH_2_ | 1.47-1.31, m  1.16-1.04, m |
| 6 |  | 44.6, CH_2_ | 3.30-3.22, m  4.33, d (13.4) |
| **phenylalanine** | | | |
| NH |  | - | 8.56, d (10.1) |
| 1 |  | 174.6^1^, C | - |
| 2 |  | 50.8, CH | 5.86, dt (10.3 7.5) |
| 3 |  | 37.9, CH_2_ | 3.55, dd (13.9, 7.3)  3.40, dd (13.8, 7.4) |
| 4 |  | 138.7, C | - |
| 5/9 |  | 130.1, CH | 7.51-7.44, m |
| 6/8 |  | 129.2, CH | 7.37-7.31, m |
| 7 |  | 127.3, CH | 7.31-7.29, m |

^1^The two carboxy carbon signals can not be assigned definitely due to resolution of the HMBC-spectrum and might be interchanged.

^2^The three carbon signals are hardly distinguishable in the ^13^C- and HSQC- as well as HMBC-spectra and might be interchanged.
